# Type 2 diabetes candidate genes, including *PAX5*, cause impaired insulin secretion in human pancreatic islets

**DOI:** 10.1172/JCI163612

**Published:** 2023-01-19

**Authors:** Karl Bacos, Alexander Perfilyev, Alexandros Karagiannopoulos, Elaine Cowan, Jones K. Ofori, Ludivine Bertonnier-Brouty, Tina Rönn, Andreas Lindqvist, Cheng Luan, Sabrina Ruhrmann, Mtakai Ngara, Åsa Nilsson, Sevda Gheibi, Claire L. Lyons, Jens O. Lagerstedt, Mohammad Barghouth, Jonathan L.S. Esguerra, Petr Volkov, Malin Fex, Hindrik Mulder, Nils Wierup, Ulrika Krus, Isabella Artner, Lena Eliasson, Rashmi B. Prasad, Luis Rodrigo Cataldo, Charlotte Ling

**Affiliations:** 1 Epigenetics and Diabetes Unit, Department of Clinical Sciences and; 2 Unit of Islet Cell Exocytosis, Department of Clinical Sciences, Lund University Diabetes Centre, Scania University Hospital, Malmö, Scania, Sweden.; 3 Endocrine Cell Differentiation, Department of Laboratory Medicine, Lund Stem Cell Center, Malmö, Scania, Sweden.; 4 Neuroendocrine Cell Biology, Department of Experimental Medical Science,; 5 Unit of Islet Pathophysiology, Department of Clinical Sciences,; 6 Human Tissue Lab, Department of Clinical Sciences,; 7 Molecular Metabolism Unit, Department of Clinical Sciences, and; 8 Genomics, Diabetes and Endocrinology, Department of Clinical Sciences, Lund University Diabetes Centre, Scania University Hospital, Malmö, Scania, Sweden.; 9 Institute of Molecular Medicine (FIMM), Helsinki University, Helsinki, Finland.; 10 The Novo Nordisk Foundation Centre for Basic Metabolic Research, Faculty of Health and Medical Sciences, University of Copenhagen, Copenhagen, Denmark.

**Keywords:** Endocrinology, Metabolism, Beta cells, Diabetes, Insulin

## Abstract

Type 2 diabetes (T2D) is caused by insufficient insulin secretion from pancreatic β cells. To identify candidate genes contributing to T2D pathophysiology, we studied human pancreatic islets from approximately 300 individuals. We found 395 differentially expressed genes (DEGs) in islets from individuals with T2D, including, to our knowledge, novel (*OPRD1*, *PAX5*, *TET1*) and previously identified (*CHL1*, *GLRA1*, *IAPP*) candidates. A third of the identified expression changes in islets may predispose to diabetes, as expression of these genes associated with HbA1c in individuals not previously diagnosed with T2D. Most DEGs were expressed in human β cells, based on single-cell RNA-Seq data. Additionally, DEGs displayed alterations in open chromatin and associated with T2D SNPs. Mouse KO strains demonstrated that the identified T2D-associated candidate genes regulate glucose homeostasis and body composition in vivo. Functional validation showed that mimicking T2D-associated changes for *OPRD1*, *PAX5*, and *SLC2A2* impaired insulin secretion. Impairments in Pax5-overexpressing β cells were due to severe mitochondrial dysfunction. Finally, we discovered PAX5 as a potential transcriptional regulator of many T2D-associated DEGs in human islets. Overall, we have identified molecular alterations in human pancreatic islets that contribute to β cell dysfunction in T2D pathophysiology.

## Graphical abstract

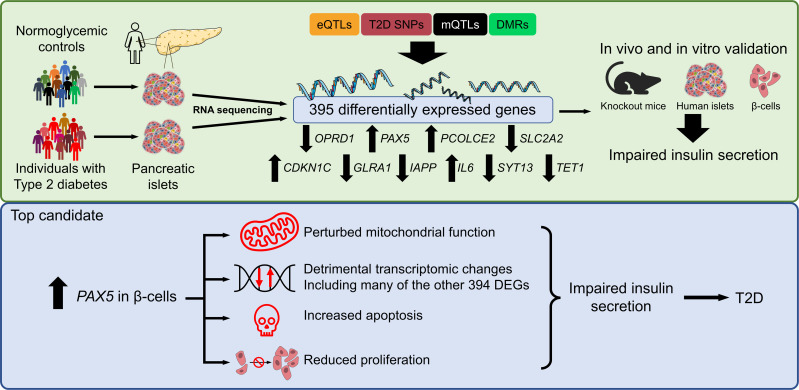

## Introduction

Type 2 diabetes (T2D) is characterized by chronic hyperglycemia due to insufficient insulin secretion from pancreatic islets, often in combination with insulin resistance in target cells. The number of individuals with T2D is increasing globally at an alarming rate, mainly due to obesity, sedentary lifestyles, and the increasing age of the world’s populations. It is therefore important to identify the molecular mechanisms that underlie the disease and to better understand why pancreatic β cells fail to adapt to an increased demand for insulin secretion, which ultimately leads to T2D.

There have been several initiatives to identify candidate genes for T2D by transcriptomic analyses in pancreatic islets from individuals with T2D and nondiabetic (ND) controls (1–9). However, most of these studies were performed in relatively small cohorts and lack replication and/or functional validation. This is especially true for single-cell RNA-Seq (scRNA-Seq) of human pancreatic islets, which has been the focus in recent years, leading to limited outcomes in terms of identifying T2D disease mechanisms (10, 11). Concurrently, GWAS have identified hundreds of genetic signals associated with T2D (12). Importantly, since many identified risk loci are associated with impaired insulin secretion and/or processes that likely affect β cells (13), these genetic studies convincingly demonstrate that pancreatic islet dysfunction is the key defect in T2D. Therefore, there is a clear need for well-powered transcriptomic analyses of human islets from individuals with T2D and ND controls. Moreover, studies functionally validating human islet transcriptomic data and further dissecting the molecular mechanisms that cause impaired insulin secretion are necessary to better understand the pathogenesis of T2D.

Here, we performed RNA-Seq of a larger set of human pancreatic islets from individuals with T2D and ND controls (in total, 309 islet preparations) to identify previously unrecognized regulators of insulin secretion that may contribute to islet dysfunction and development of T2D. To evaluate whether the identified changes may influence disease development, we further tested whether a predictor of T2D (14), hemoglobin A1c (HbA1c) levels, associated linearly with islet expression of the identified genes in individuals not diagnosed with T2D. We next investigated which cell type or types expressed the identified genes. Data from assay for transposase-accessible chromatin with high-throughput sequencing (ATAC-Seq), DNA methylation analysis, GWAS, and the International Mouse Phenotyping Consortium (IMPC) were then analyzed to further dissect the regulation and function of identified genes. T2D candidate genes not identified in previous studies were then manipulated in human islets or clonal β cells to explore whether they directly impact insulin secretion. Finally, we investigated whether the transcription factor PAX5, our main T2D candidate, is a master regulator of the identified differentially expressed genes (DEGs) in human pancreatic islets.

## Results

### Transcriptomic analysis of human pancreatic islets identified T2D candidates.

**Figure 1:**
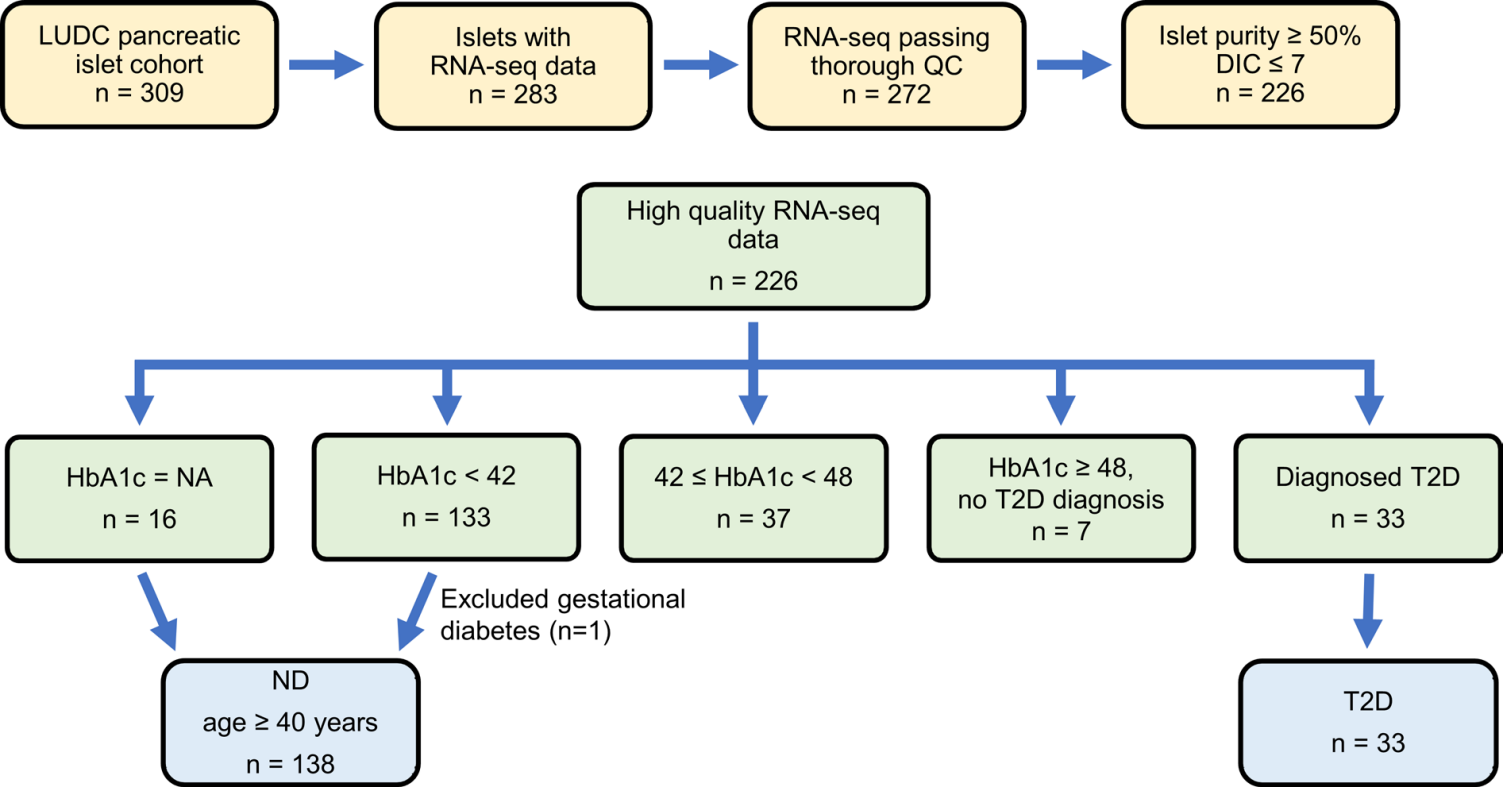
Workflow of RNA-Seq sample filtering. The LUDC pancreatic islet cohort consists of islet preparations from 309 individuals. RNA-Seq was performed on 283 of these. After quality control (QC) and other filtering, data from 171 preparations were included in the analysis to identify DEGs in islets from individuals with T2D versus ND control islets. Similarly, islet data from 176 preparations were included in the HbA1c analysis.

To identify regulators of insulin secretion that may contribute to T2D, we generated RNA-Seq data from the large Lund University Diabetes Centre (LUDC) pancreatic islet cohort, comprising islets from a total of 309 donors (Supplemental Table 1; supplemental material available online with this article; https://doi.org/10.1172/JCI163612DS1). From these, RNA from 283 islet samples was successfully sequenced (Figure 1). Before further analysis and to ensure the highest possible quality of data, we filtered our islet samples (Figure 1). ND controls were defined as individuals lacking a diabetes diagnosis and exhibiting HbA1c levels below 42 mmol/mol. Moreover, age of 40 years or older was used as an inclusion criterion for the ND controls, since none of the individuals with T2D were younger than 40 years of age. The characteristics of the individuals in the LUDC islet case-control cohort are summarized in Table 1 and Supplemental Figure 1, A and B. We subsequently compared RNA-Seq data from islets from 138 ND controls with those from 33 individuals with T2D (Figure 1) using a generalized linear model with correction for age, sex, islet purity, and days in culture (DIC). The analysis identified 395 DEGs (FDR below 5%, *q* < 0.05, Supplemental Table 2, sheet A), which included 228 upregulated and 167 downregulated genes. We further applied a model in which we also added BMI as a covariate; expression of 394 of the 395 DEGs was then associated with T2D (*P* = 8.6 × 10^–17^ to 4.5 × 10^–2^, Supplemental Table 2, sheet A). Additionally, we performed an analysis including only donors with an islet purity of 80% or higher (*n* = 69 ND and *n* = 18 T2D); expression of 334 of 395 DEGs was then associated with T2D (*P* = 6.7 × 10^–11^ to 4.9 × 10^–2^, Supplemental Table 2, sheet A).

**Figure 2:**
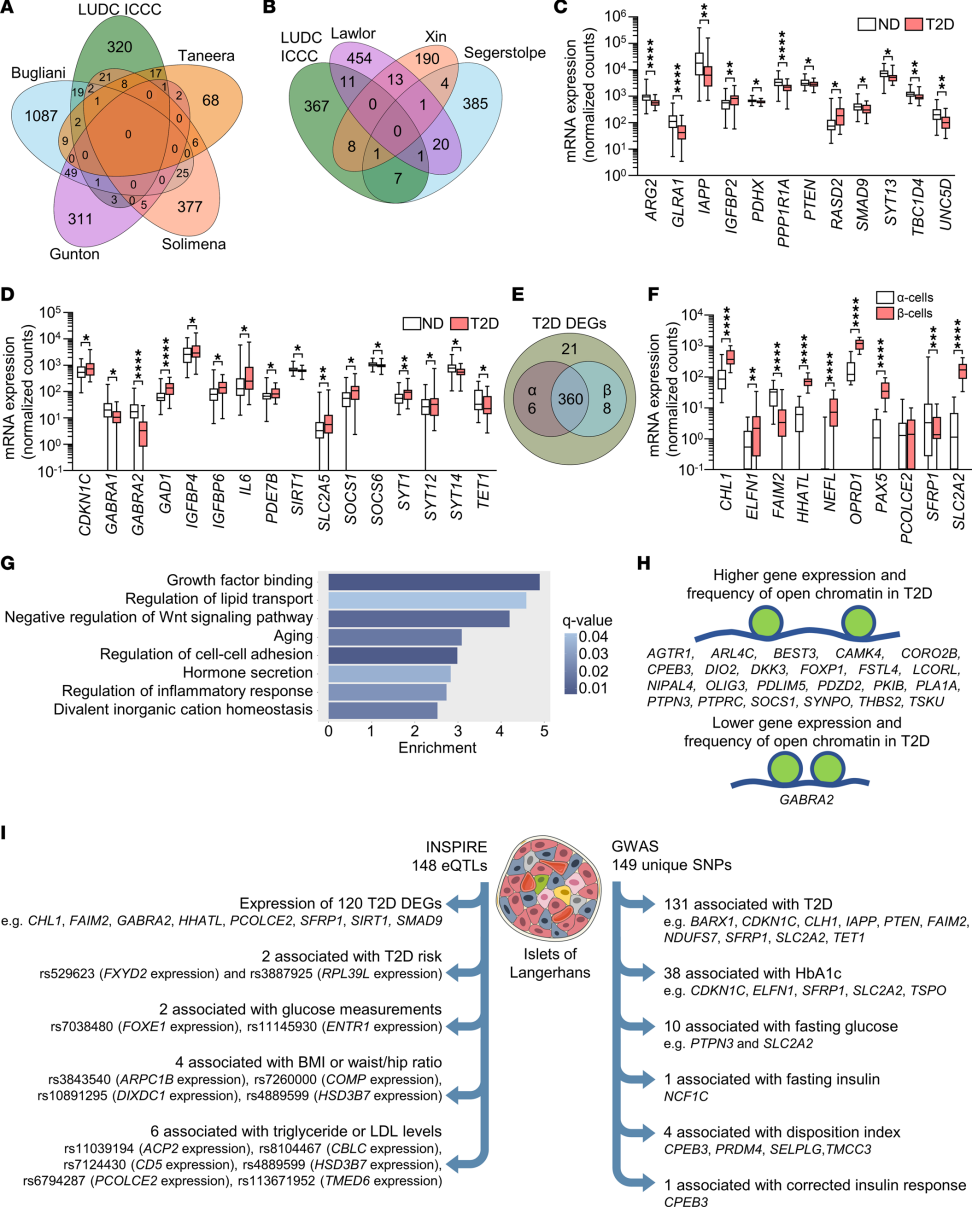
Characterization of the 395 identified DEGs. (**A** and **B**) Venn diagrams showing the overlaps between the DEGs identified in the LUDC islet case-control cohort (LUDC ICCC) and DEGs identified in previous bulk (**A**) and single-cell (**B**) expression analyses of human pancreatic islets from T2D and ND donors. (**C** and **D**) mRNA expression of selected known (**C**) and, to our knowledge, novel (**D**) DEGs identified in pancreatic islets from 33 individuals with T2D and 138 ND controls of the LUDC ICCC. *q < 0.05, **q < 0.01, and ****q < 0.0001, based on a generalized linear model as implemented in DESeq2 (70), with correction for age, sex, islet purity, and DIC. (**E**) RNA-Seq of sorted α and β cells from 16 ND individuals showed that the vast majority of the 395 identified DEGs were expressed in either or both cell types. (**F**) mRNA expression of selected genes in sorted α and β cells from islet preparations from 16 ND individuals. **q < 0.01, ***q < 0.001, and ****q < 0.0001, based on a generalized linear model as implemented in DESeq2 (70). (**G**) Enrichment analysis showed that T2D islet DEGs were enriched for gene ontology terms associated with β cell function. (**H**) DEGs with an altered chromatin state in islets from ND controls versus individuals with T2D, as identified by ATAC-Seq (15). (**I**) Left: 148 pancreatic islet eQTLs associated with expression of 120 DEGs as well as T2D risk and metabolic traits. Right: 149 unique SNPs annotated to 106 DEGs were found to associate with T2D or the indicated glucose traits in GWAS. Box-and-whisker plots show the median, 25th and 75th percentiles, and minimum and maximum values. Illustration credit for the islet in Figure 2I: Servier Medical templates.

For replication, we next compared our 395 identified DEGs with published expression data from studies of human pancreatic islets from T2D case-control cohorts (1–3, 5–8). Previous bulk expression studies identified 75 of the 395 DEGs identified here (Figure 2A and Supplemental Tables 2 and 3) (1, 2, 6, 7). Additionally, published scRNA-Seq studies found differential expression of 28 of our 395 DEGs in islet cells from ND controls versus individuals with T2D (Figure 2B and Supplemental Tables 2 and 3) (3, 5, 8). Eight and 9 of these DEGs were differentially expressed in α and β cells, respectively (Supplemental Table 2, sheet A), and their expression in islets is presented in Supplemental Figure 1, C and D. Taken together, 94 of our 395 DEGs have been identified in one or more of these previous studies, whereas 301 were not (Supplemental Table 2, sheet A). *ARG2*, *GLRA1*, *IAPP*, *IGFBP2*, *PDHX*, *PPP1R1A*, *PTEN*, *RASD2*, *SMAD9*, *SYT13*, *TBC1D4*, and *UNC5D* are DEGs previously identified by the studies included in Figure 2, A and B (Figure 2C and Supplemental Table 2, sheet A), while, for example, *CDKN1C*, *GABRA1*, *GABRA2*, *GAD1*, *IGFBP4*, *IGFBP6*, *IL6*, *PDE7B*, *SIRT1*, *SLC2A5*, *SOCS1*, *SOCS6*, *SYT1, SYT12, SYT14*, and *TET1* are DEGs not identified by those studies (Figure 2D and Supplemental Table 2, sheet A).

To better understand which islet cell types expressed our 395 DEGs, we studied their expression in FACS human α and β cells from the LUDC sorted α/β cell cohort. This cohort included 13 ND individuals, 2 individuals with T2D, and 3 individuals with prediabetes (Table 1). We found that α and β cells expressed 366 and 368 of our DEGs, respectively (Figure 2E and Supplemental Table 2, sheet A). Of these, 90 genes showed higher expression in α versus β cells, while 174 had higher expression in β versus α cells (*q* < 0.05, Supplemental Table 2, sheet A). This included 9 of the 11 genes that we selected for functional validation (see below and Figure 2F). Using WebGestaltR, we found 17 enriched gene sets (*q* < 0.05), including divalent inorganic cation homeostasis and positive regulation of cell adhesion, among the DEGs with higher expression in α versus β cells (Supplemental Figure 1E), while there was no enrichment among the DEGs with higher expression in β versus α cells. We also examined the expression of our DEGs in different islet cell types using published human islet scRNA-Seq data (5). We found that among our 395 DEGs, 366 (93%) were expressed in α cells, 347 (88%) in β cells, 331 (84%) in pancreatic polypeptide (PP) cells, 312 (79%) in delta (δ) cells, and 190 (48%) in epsilon (ε) cells (Supplemental Table 4 and Supplemental Figure 2). We found that 97%–98% of DEGs expressed in α and β cells in the scRNA-Seq expression data overlapped with those expressed in α and β cells from the LUDC sorted α/β cell cohort. We further tested whether any of the 395 islet DEGs also exhibited differential expression in the same direction in sorted α or β cells when comparing cells from 5 donors with T2D or prediabetes versus those from 13 ND controls (Table 1). Here, 4 and 28 of our 395 DEGs showed differential expression (*P* < 0.05) in sorted α or β cells, respectively (Supplemental Table 2, sheets B and C, Supplemental Figure 3, A and B). The DEGs in sorted β cells included *BARX1*, *NEFL*, *PAX5*, and *PCOLCE2*, genes we later selected for functional follow-up (see below). It should be noted that, given the modest sample size, there was limited power when comparing T2D with ND samples from both the scRNA-Seq and sorted RNA-Seq data, and hence one would not expect to find all 395 DEGs in these analyses. These data suggest that several DEGs in T2D islets may affect insulin secretion or other aspects of β cell function.

Our data clearly show that individuals with T2D exhibit abundant transcriptomic alterations in pancreatic islets. However, it is not clear whether these changes predispose individuals to T2D or whether they are a consequence of disease progression. To determine whether changes in expression levels of the 395 identified DEGs potentially predispose individuals to T2D, we studied their expression in human islets from the LUDC islet HbA1c cohort (Table 1), including individuals with a wide range of HbA1c levels who were not previously diagnosed with diabetes. Interestingly, expression of 142 of the 395 DEGs (36%) was linearly associated with HbA1c levels (Supplemental Table 2, sheet A). Among those genes, all had a β coefficient with the same directionality as that of the expression differences seen in islets from the LUDC islet case-control cohort. These data suggest that the changes in expression may occur before the diagnosis of T2D and potentially contribute to the development of the disease. They also indicate that the expression changes were not due to T2D treatment.

### Gene sets, including hormone secretion, are enriched among T2D-associated DEGs.

We used WebGestaltR and gene ontology to discover enriched gene sets among our 395 DEGs. We found 39, 5, and 6 enriched gene sets for biological processes, cellular components, and molecular functions, respectively (*q* < 0.05, Supplemental Table 5). Interestingly, gene sets for growth factor binding, regulation of lipid transport, negative regulation of Wnt signaling, aging, regulation of cell-cell adhesion, hormone secretion, regulation of inflammatory response, and divalent inorganic cation homeostasis were among the significantly enriched gene sets (Figure 2G).

### T2D-associated DEGs display alterations in open chromatin and DNA methylation in human islets.

Open chromatin regions are associated with active gene transcription (15). Of our 395 DEGs in islets from ND controls versus those from individuals with T2D, 346 (88%) were marked by at least 1 open chromatin region, identified by previous ATAC-Seq of human pancreatic islets (15). From this subset, 194 and 152 DEGs were upregulated and downregulated, respectively, in islets from individuals with T2D. The top DEGs *BARX1*, *OPRD1*, *PAX5*, and *PCOLCE2* overlapped with several open chromatin regions (4, 4, 5, and 6, respectively) located both upstream and downstream of the respective transcription start sites (Supplemental Table 6, sheet A). Moreover, 24 DEGs, including *CAMK4*, *DIO2,* *DKK3*, *FOXP1*, *GABRA2*, *PTPRC*, *SOCS1*, and *SYNPO*, displayed alterations of open chromatin in islets from ND controls versus those from individuals with T2D. Of these, 23 exhibited more open chromatin and higher expression, and 1 (*GABRA2*) showed less open chromatin and reduced expression, in islets from donors with T2D (Figure 2H, Supplemental Table 6, sheet A). Together, these data suggest an altered chromatin state in islets from individuals with T2D that was associated with altered gene expression for a subset of identified DEGs.

We further tested whether the 395 DEGs showed altered DNA methylation in islets from individuals with T2D versus those from ND controls based on published data (16) and found 732 differentially methylated regions (DMRs) annotated to 262 DEGs (Supplemental Table 6, sheet B). These included DMRs annotated to *CHL1*, *ELFN1, FAIM2*, *HHATL*, *OPRD1*, *PAX5*, *SFRP1*, and *SLC2A2,* DEGs selected for functional follow-up (see below).

### SNPs associate with the identified DEGs, T2D, and metabolic traits.

We next assessed whether SNPs were associated with expression of the 395 DEGs. We first explored expression quantitative trait loci (eQTL) data from human pancreatic islets from the INSPIRE (Integrated Network for Systematic analysis of Pancreatic Islet RNA Expression) study (17) and the T2D Genes portal (https://t2d.hugeamp.org/). We found 148 SNPs associated with islet expression of 120 of our 395 DEGs, including *CHL1*, *FAIM2*, *GABRA2*, *HHATL*, *PCOLCE2*, *SFRP1*, *SIRT1*, and *SMAD9* (Supplemental Table 7, sheet A, and Figure 2I). We then used the GWAS catalog to determine whether any of these 148 SNPs have previously been linked to T2D or other metabolic diseases or traits (Supplemental Table 7, sheet A). Indeed, 2 eQTL SNPs associated with *FXYD2* (rs529623) and *RPL39L* (rs3887925) islet expression have been linked to T2D risk (12) (Supplemental Table 7, sheet A, and Figure 2I). Moreover, 2 eQTL SNPs associated with islet expression of *FOXE1* (rs7038480) and *ENTR1* (rs11145930) have been linked to blood glucose levels (12, 18–20) (Supplemental Table 7, sheet A, and Figure 2I). Four eQTL SNPs associated with islet expression of *ARPC1B* (rs3843540), *COMP* (rs7260000), *DIXDC1* (rs10891295), and *HSD3B7* (rs4889599) have been linked to BMI and/or waist-to-hip ratio (18, 21) (Supplemental Table 7, sheet A, and Figure 2I). Finally, 6 eQTL SNPs associated with islet expression of *ACP2* (rs11039194), *CBLC* (rs8104467), *CD5* (rs7124430), *HSD3B7* (rs4889599), *PCOLCE2* (rs6794287), and *TMED6* (rs113671952) have been linked to triglyceride or LDL cholesterol levels (18, 22–24) (Supplemental Table 7, sheet A, and Figure 2I). These data demonstrate that SNPs associated with islet expression of numerous identified DEGs impact the risk for T2D and metabolic traits (Figure 2I).

As our islet cohort data included variables, e.g., islet purity, that were not available or used in the INSPIRE study (17), and since we used a cutoff for islet purity, we also performed an eQTL analysis adjusting for age, sex, BMI, DIC, purity, and T2D in the LUDC islet case-control cohort. This showed that 26 of our 395 DEGs, including *BEST3*, *HSD3B7*, and *TMED6*, had at least 1 eQTL, based on a *q* value of less than 0.05. Moreover, 374 DEGs had eQTLs with nominal *P* values (*P* < 0.05, Supplemental Table 7, sheet B), and these included 47 of the 148 SNPs and 117 of the 123 genes identified in the INSPIRE analysis (Supplemental Table 7, sheets A and B).

We then assessed whether SNPs that map to any of the 395 DEGs have been associated with T2D and/or glycemic traits (HbA1c, fasting glucose, fasting insulin, homeostatic model assessment of β cell function [HOMA-B], disposition index [DI], or corrected insulin response [CIR]) in GWAS using the Common Metabolic Diseases Knowledge Portal (hugeamp.org, accessed August 2022). A total of 106 DEGs were linked to 149 unique SNPs associated with these traits in GWAS (Supplemental Table 7, sheet C, Figure 2I); 131 SNPs were associated with T2D, 38 with HbA1c, 10 with fasting glucose, 1 with fasting insulin, 4 with DI, and 1 with CIR. This included 1 SNP mapping to each of *BARX1*, *CHL1*, *ELFN1*, and *FAIM2*, three SNPs mapping to *SLC2A2*, and 4 SNPs mapping to *SFRP1*, DEGs we subsequently selected for functional follow-up (see below).

Since genetic and epigenetic mechanisms interact and together affect biological processes, we tested whether SNPs in *cis* are associated with DNA methylation of CpG sites annotated to the 395 DEGs in human pancreatic islets, so-called mQTLs (25). We found 490 SNPs associated with DNA methylation of 176 unique sites annotated to 90 DEGs (Supplemental Table 7, sheet D). These included mQTLs, 3 each, annotated to *OPRD1* and *PAX5*, two additional DEGs we subsequently selected for functional follow-up (see below).

Panels A–C in Supplemental Figure 4 show the overlap between DEGs that had eQTLs, DMRs, GWAS SNPs, ATAC-Seq peaks, or mQTLs annotated, and the overlap between the SNPs in the different genetic analyses above.

### The identified T2D-associated DEGs affect metabolism in vivo.

**Figure 3:**
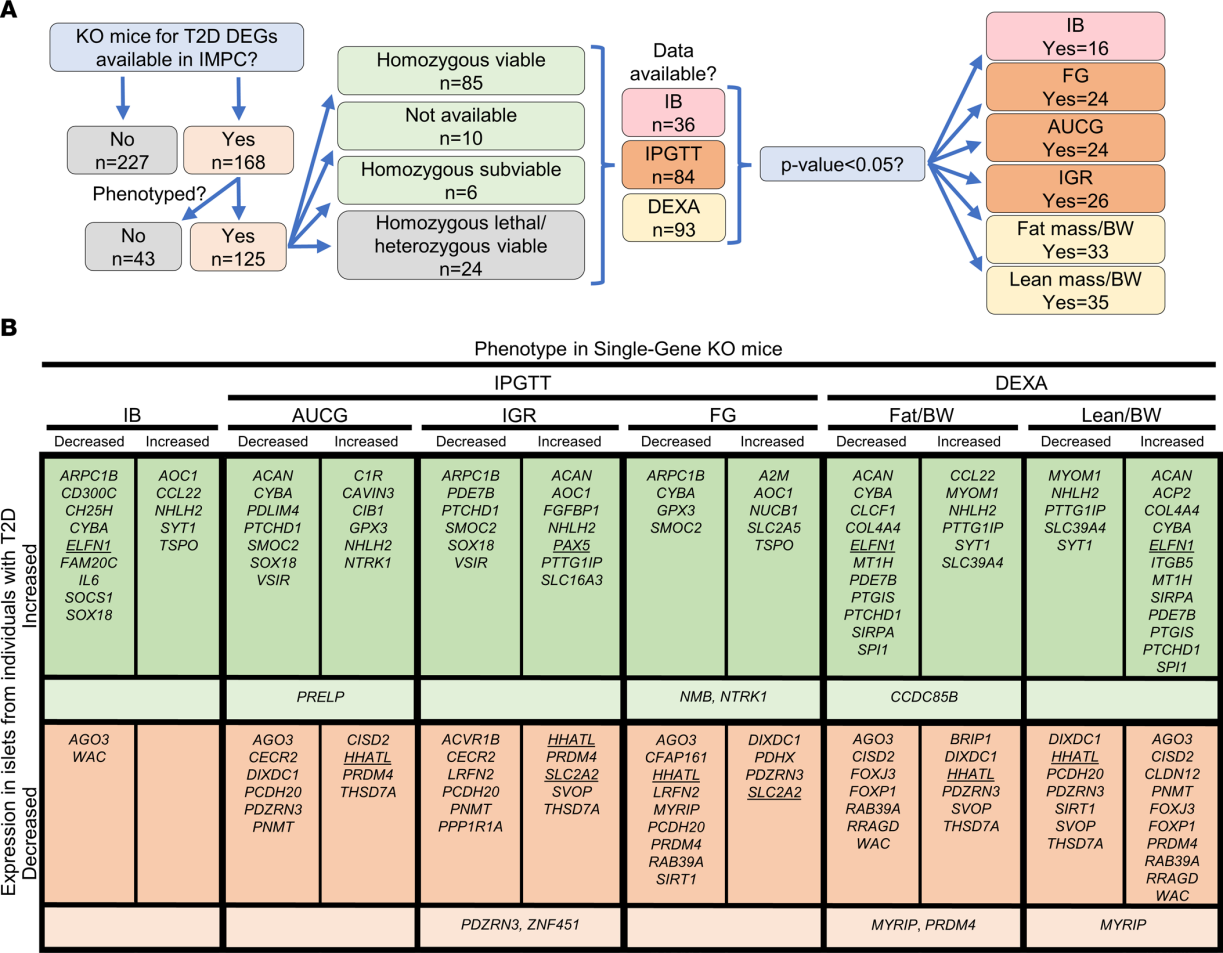
Mice with KO of genes showing differential expression in pancreatic islets from individuals with T2D exhibit metabolic phenotypes. (**A**) Flow chart depicting the IMPC data-mining strategy and an overview of the findings for mice with KO of DEGs identified in the LUDC islet case-control cohort. (**B**) Summary of IMPC phenotypic data outputs for viable KO mouse strains. Underlined genes were functionally validated in our study, while KO mice for genes in lighter-colored areas show different effects for the indicated phenotype in males and females. IB, insulin blood levels.

To explore potential in vivo evidence for protective or susceptible functions in the development of diabetes for the 395 identified T2D DEGs, we systematically searched the IMPC database (https://www.mousephenotype.org/, accessed April 15, 2020). The IMPC database contains KO mouse strain entries for 168 of the 395 DEGs, with phenotype data available for 125 strains (Figure 3A). Most were homozygous viable (*n* = 85), with fewer that were homozygous subviable (meaning that the number of alive homozygous pups was lower than expected, *n* = 6), homozygous lethal but heterozygous viable (*n* = 24), or of unknown viability (*n* = 10). Supplemental Table 8 lists DEGs found in T2D islets with IMPC metabolic readouts for KO strains (*n* = 125). Of the phenotyped KO strains, insulin blood (IB) measurement data were available for 36 KO strains, with 16 (44%) displaying altered levels (*P* < 0.05, Figure 3 and Supplemental Table 8). Intraperitoneal glucose tolerance test (IPGTT) data showed that the fasted blood glucose concentration (FG) and AUC for glucose (AUCG) were altered in 24 of 81 (30%) and 24 of 83 (29%) KO strains, respectively (*P* < 0.05), while the initial response to glucose (IGR) challenge was altered in 26 of 80 (33%) (*P* < 0.05) (Figure 3 and Supplemental Table 8). With respect to body composition, dual-energy x-ray absorptiometry (DEXA) phenotypic data were available for 93 KO models, and among these, 33 (35%) and 35 (38%) had altered total fat mass and total lean mass, respectively (*P* < 0.05, Figure 3 and Supplemental Table 8).

The effects of gene KO on the studied metabolic phenotypes agreed with the direction of altered expression for several of the T2D-associated DEGs. For example, our data show that *HHATL* expression was significantly decreased in T2D islets (Supplemental Table 2). In line with this, IMPC data showed that *Hhatl*-KO mice had impaired glucose tolerance, increased total body fat mass, and decreased total body lean mass (Figure 3B). *SLC2A2* expression was also decreased in islets from individuals with T2D (Supplemental Table 2), and *Slc2a2-*KO mice had increased FG levels and IGR. Overall, these rodent in vivo findings indicate an important role for the identified DEGs in the control of glucose homeostasis and body composition.

### Functional follow-up shows that changes in expression of T2D-associated DEGs impair β cell function.

**Figure 4:**
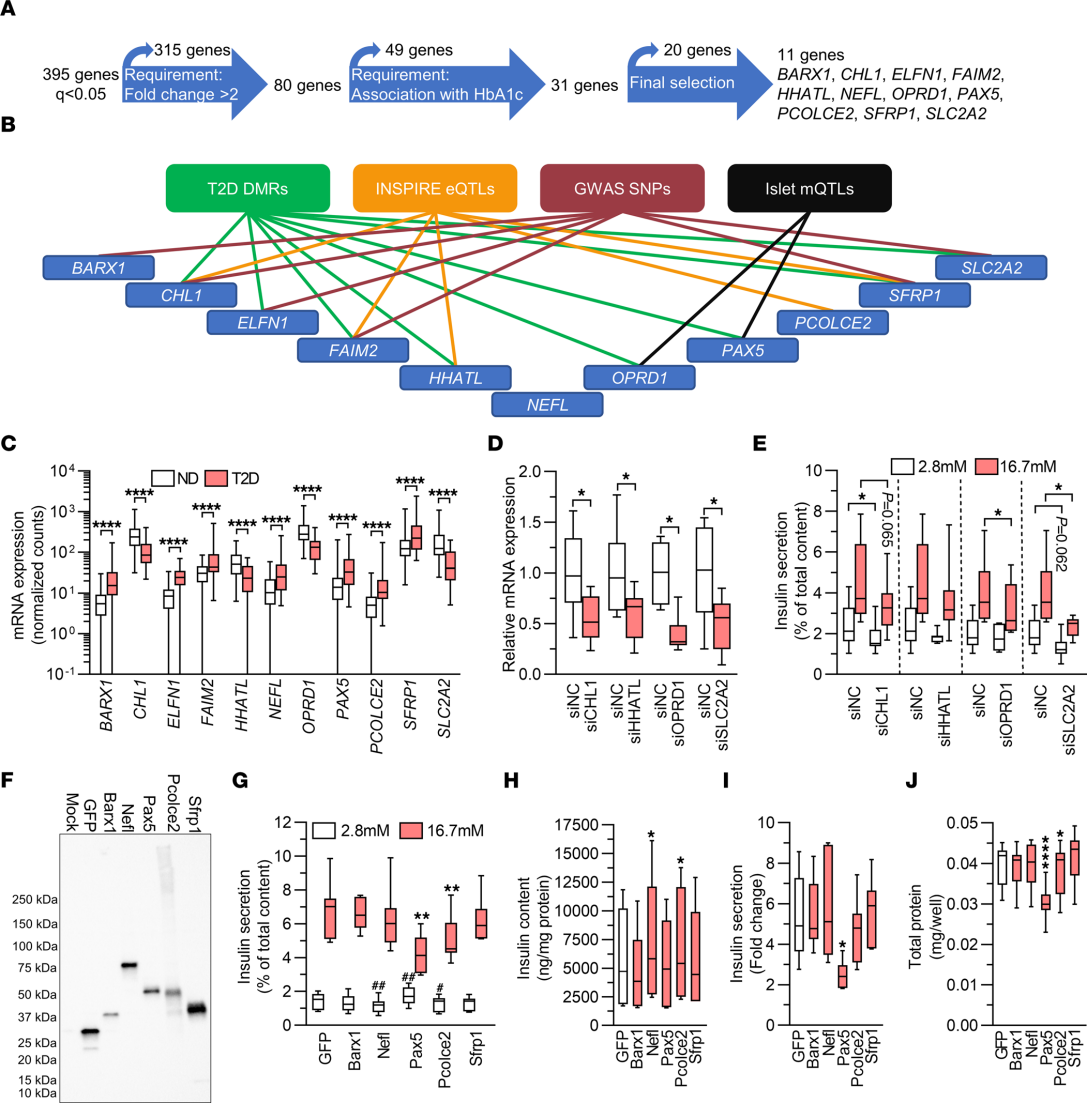
T2D-associated expression changes impair insulin secretion. (**A**) Flow chart showing strategy for the selection of DEGs for functional follow-up. (**B**) Ten of the 11 genes selected for functional analysis have T2D-associated DMRs, INSPIRE eQTLs, SNPs associated with T2D or glucose traits, or islet mQTLs annotated to them. (**C**) mRNA expression of DEGs selected for functional follow-up. ****q < 0.0001, based on a generalized linear model as implemented in DESeq2 (70), with correction for age, sex, purity, and DIC, on expression data on islets from 138 ND controls and 33 individuals with T2D. (**D**) qPCR quantification of siRNA-mediated knockdown of *CHL1*, *HHATL*, *OPRD1*, and *SLC2A2* in human islets (*n* = 6–8). (**E**) Effect of knockdown of *CHL1*, *HHATL1*, *OPRD1*, or *SLC2A2* on insulin secretion from human islets (*n* = 6–8). (**D** and **E**) *P < 0.05 compared with negative control siRNA (siNC) at the indicated glucose concentration; 2-tailed, paired *t* test. (**F**) Representative Western blot showing overexpression of GFP, Barx1, Nefl, Pax5, Pcolce2, and Sfrp1 in virally transduced INS1 β cells. The experiment was performed 3 times. (**G**–**J**) Effect of overexpression on insulin secretion (**G**) and insulin content (**H**) in absolute values, insulin secretion presented as fold change (**I**), and total protein (**J**) (*n* = 7). (**G**) **P < 0.01 compared with GFP at 16.7 mM glucose; ^#^*P* < 0.05 and ^##^*P* < 0.01 compared with GFP at 2.8 mM glucose. (**H**–**J**) *P < 0.05 and ****P < 0.0001 compared with GFP. Data in **G**–**J** were analyzed by 2-tailed, paired *t* test. Box-and-whisker plots show the median, 25th and 75th percentiles, and minimum and maximum values.

We then asked whether any DEGs previously unrecognized in comparable studies have a functional role in β cells. First, we used a strategy to select genes for functional investigation (Figure 4A). We selected DEGs with a greater than 2-fold change in either direction, based on mean expression levels in islets in ND controls versus individuals with T2D, and the same directionality of change in expression as the HbA1c correlation in islets from the LUDC islet HbA1c cohort. This generated a list of 31 genes. We further excluded DEGs that were not expressed in the sorted α or β cells from ND controls, unless their expression was induced in islets from individuals with T2D, and that were not expressed in endocrine cell types in the scRNA-Seq data sets. Finally, on the basis of the lowest *q* values, we selected the top 9 genes that, to our knowledge, had not previously been studied in β cells (Figure 4A). In addition, we included the genes *CHL1* and *SLC2A2*. Knockdown of *Chl1* in clonal β cells was previously found to impair glucose-stimulated insulin secretion (GSIS) (26), and we included this gene as a positive control. *SLC2A2,* encoding the glucose transporter GLUT2, has also been studied, but we included it as it is debated whether its function is significant in human β cells (27, 28). Of note, 10 of these 11 genes have either T2D-associated DMRs (Supplemental Table 6, sheet B), INSPIRE eQTLs (Supplemental Table 7, sheet A), SNPs in *cis* associated with T2D and/or glycemic traits (Supplemental Table 7, sheet C), or mQTLs (Supplemental Table 7, sheet D), as summarized in Figure 4B. Moreover, all 11 DEGs have nominal LUDC eQTLs (*P* = 2.7 × 10^–4^ to 2.3 × 10^–2^, Supplemental Table 7, sheet B).

To mimic the situation in individuals with T2D, the selected genes that had lower expression levels in islets in T2D, i.e., *CHL1*, *HHATL*, *OPRD1*, and *SLC2A2* (Figure 4C), were silenced by siRNA transfection in human islets from ND individuals. This resulted in a 50%–60% reduction in gene expression (Figure 4D). Determinations of insulin secretion showed that islets deficient for *CHL1* displayed a reduction in insulin release when exposed to basal glucose (2.8 mM), whereas islets deficient for *SLC2A2* showed nominally reduced insulin secretion at the same glucose concentration (*P* = 0.062, Figure 4E). At stimulatory glucose levels (16.7 mM), *OPRD1* or *SLC2A2* silencing resulted in approximately 20% and 40% reduced insulin secretion, respectively, while islets deficient for *CHL1* or *HHATL* showed nominally reduced and unaffected secretion, respectively (Figure 4E). The changes did not translate into significant differences in the fold change of insulin secretion (secretion at stimulatory glucose divided by secretion at basal glucose levels) and occurred without differences in insulin content (Supplemental Figure 5, A and B). We also measured glucagon secretion from islets in which *CHL1*, *HHATL*, *OPRD1*, or *SLC2A2* had been silenced and found no differences (Supplemental Figure 5C). These data indicate that reduced islet expression of *OPRD1* and *SLC2A2* may contribute to the insulin secretion defect seen in individuals with T2D.

Selected DEGs that exhibited higher islet expression in T2D, i.e., *BARX1*, *ELFN1*, *FAIM2*, *NEFL*, *PAX5*, *PCOLCE2*, and *SFRP1* (Figure 4C), were overexpressed by plasmid transfection in 832/13 INS1 β cells (hereafter called INS1 β cells). We used INS1 β cells for overexpression experiments because of the limited supply of human islets. Furthermore, in our hands, INS1 β cells behaved more like primary mature human β cells than did the fetal human β cell line EndoC-βH1 in gene manipulation experiments (KB and JKO, unpublished observations). Plasmid transfection resulted in impaired insulin secretion in absolute numbers at basal or stimulatory glucose levels, reduced insulin secretion expressed as fold change, or altered insulin content in cells overexpressing *Barx1*, *Nefl*, *Pax5*, *Pcolce2*, or *Sfrp1* (Supplemental Figure 5, D–G). However, the plasmid transfection efficiency was low (20%–30% of cells, Supplemental Figure 5, H–J). This, together with the fact that expression was driven by the very strong CMV promoter, made us wary that the phenotypes may be due to very high overexpression overwhelming the transfected cells. We therefore proceeded to use lentiviral vectors, allowing for transgene delivery to a larger proportion of cells, with the weaker human phosphoglycerate kinase 1 promoter driving expression of HA-tagged *Barx1*, *Nefl*, *Pax5*, *Pcolce2*, or *Sfrp1*. Western blot analysis showed that transduction of INS1 β cells with these vectors resulted in expression of proteins of expected sizes (Figure 4F). Overexpression of Pax5 significantly increased basal secretion and strongly reduced insulin secretion at stimulatory glucose levels, without influencing insulin content (Figure 4, G and H), whereas Nefl or Pcolce2 overexpression caused reduced insulin secretion at basal (both) and stimulatory (Pcolce2) glucose levels (Figure 4G) and increased insulin content (Figure 4H). Overexpression of Barx1 or Sfrp1 did not alter insulin secretion or content. The changes described caused a strong reduction in fold change of insulin secretion in Pax5-overexpressing INS1 β cells (Figure 4I). Finally, wells where cells were transduced with the Pax5 or Pcolce2 viruses also contained significantly less total protein than wells with cells transduced with the GFP virus (Figure 4J). This indicates a lower cell number and, hence, suggests that Pax5 and Pcolce2 may affect cell viability and/or proliferation and therefore potentially β cell mass in T2D. Taken together, these functional experiments identified 4, to our knowledge, previously unrecognized regulators of insulin secretion showing either lower (*OPRD1*) or higher (*NEFL*, *PAX5*, and *PCOLCE2*) expression in islets from individuals with T2 (Figure 4, C–J). Because Pax5 overexpression had the strongest effect on both insulin secretion and protein content (Figure 4, G and J), we decided to further explore its impact on β cells. First, to exclude the possibility that the HA tag renders Pax5 pathogenic, we repeated the secretion experiments with viral vectors conferring expression of untagged Pax5. The results showed that also untagged Pax5 reduced GSIS in INS1 β cells (Supplemental Figure 5K).

### Overexpression of Pax5 perturbs mitochondrial function and causes β cell loss.

**Figure 5:**
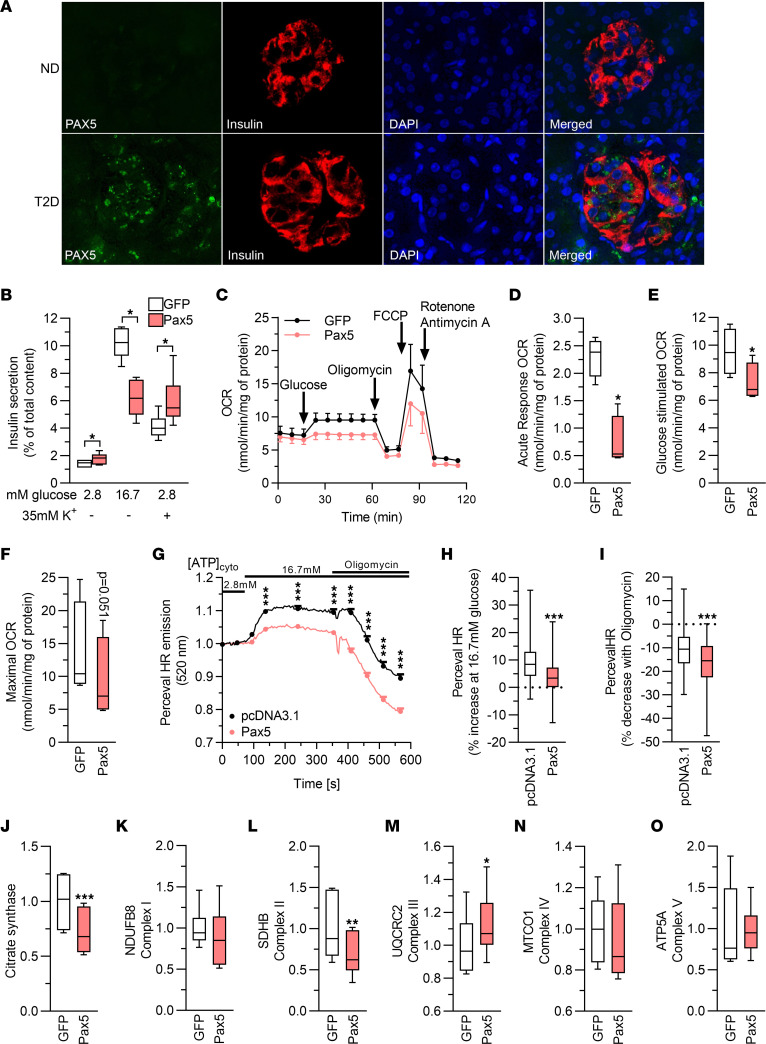
Increased expression of Pax5 results in perturbed mitochondrial activity. (**A**) Immunohistochemical staining of human pancreas sections (*n* = 5 ND; *n* = 4 T2D) showing increased PAX5 (green) expression in T2D pancreas sections. Most of the expression was confined to β cells, as evidenced by costaining with insulin (red). Nuclei are stained with DAPI (blue). Representative images are shown (original magnification, ×40). (**B**) Pax5 overexpression blunted GSIS but increased secretion stimulated by elevated K^+^ (*n* = 6). (**C**) OCR in clonal β cells overexpressing GFP or Pax5. The OCR was measured at 2.8 mM glucose (basal respiration) and then after sequential addition of 16.7 mM glucose (glucose-stimulated respiration), 5 μM oligomycin (inhibits ATP synthase), 4 μM carbonyl cyanide *p*-trifluoromethoxyphenylhydrazone (FCCP, mitochondrial uncoupler), and 1 μM rotenone/antimycin A (electron transport chain inhibitors) (*n* = 4). (**D**–**F**) Respiratory response to addition of high glucose (change in respiration compared with basal glucose, **D**), glucose-stimulated respiration (respiration at high glucose, **E**), and maximal OCR (respiration after mitochondrial uncoupling, **F**) (*n* = 4). (**G**) PercevalHR trace on INS1 β cells stimulated with 2.8 and 16.7 mM glucose, and after addition of oligomycin (*n* = 148, pcDNA3.1; *n* = 213, Pax5). (**H** and **I**) Pax5-overexpressing INS1 β cells (*n* = 213) exhibited a significantly lower increase of the ATP/ADP ratio when glucose was raised to 16.7 mM (average signal between 94 and 354 seconds compared with average signal between 0 and 75 seconds, **H**), and a greater drop in the ATP/ADP ratio after addition of oligomycin (average signal between 409 and 567 seconds compared with average signal between 94 and 354 seconds, **I**), when compared with pcDNA3.1-transfected cells (*n* = 148). (**J**–**O**) Levels of citrate synthase and subunits of complex I–V of the electron transport chain (*n* = 6). *P < 0.05, **P < 0.01, and ***P < 0.001, by 2-tailed, paired *t* test (**B**–**F** and **J**–**O**) and 2-tailed, unpaired *t* test (**G**–**I**). Box-and-whisker plots show the median, 25th and 75th percentiles, and minimum and maximum values.

*PAX5* encodes a transcription factor associated with leukemia (29) and, to our knowledge, has not been studied in β cells. First, we used IHC to assess the distribution of PAX5 in human islets. In accordance with the RNA-Seq data, we found that islets from individuals with T2D displayed robust PAX5 expression, with most of the staining found in β cells, whereas PAX5 was barely detectable in islets from ND controls (Figure 5A).

To further characterize the Pax5-induced defects in INS1 β cells, we stimulated insulin secretion with a depolarizing concentration of K^+^. These experiments again showed that GSIS was severely blunted by Pax5 overexpression, while K^+^-stimulated secretion was increased (Figure 5B). This indicates that secretory defects induced by Pax5 overexpression occurred before depolarization of K_ATP_ channels in the insulin secretion pathway, potentially in mitochondrial metabolism. To determine whether Pax5 overexpression alters mitochondrial metabolism in β cells, we used the Seahorse XF analyzer to measure the oxygen consumption rate (OCR). This showed that Pax5 overexpression caused a reduction in mitochondrial respiration with significantly lower glucose-stimulated respiration, both in terms of increase compared with basal respiration and of absolute values, as well as lower maximal respiration (Figure 5, C–F). These respiratory defects were reflected in a reduced PercevalHR signal, demonstrating a lower ATP/ADP ratio in response to glucose stimulation in Pax5-overexpressing β cells (Figure 5, G–I). To find a possible cause for these mitochondrial defects, we performed Western blotting to measure protein levels of subunits of complex I–V in the electron transport chain and of the citric acid cycle enzyme citrate synthase. We found that the levels of citrate synthase and Sdhb (complex II) were reduced by Pax5 overexpression, while Uqcrc2 (complex III) was slightly increased (Figure 5, J–O, and Supplemental Figure 5, L and M). Together, these data support the idea that increased Pax5 levels perturb insulin secretion by negative effects on mitochondrial function.

**Figure 6:**
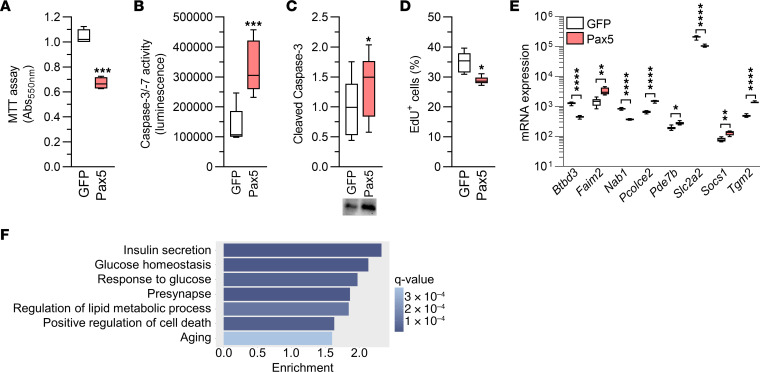
Elevated Pax5 in INS1 β cells leads to cell loss and widespread transcriptomic changes affecting β cell function. (**A**) Pax5 overexpression resulted in loss of INS1 β cells, as indicated by MTT assay (*n* = 4). (**B** and **C**) Pax5 overexpression in INS1 β cells increased caspase-3/-7 activity (*n* = 5) (**B**) and levels of cleaved (i.e., active) caspase-3 (*n* = 6, all samples were run on 1 gel) (**C**). (**D**) Pax5 overexpression reduced proliferation in INS1 β cells (*n* = 6). (**E**) mRNA expression of *Btbd3*, *Faim2*, *Nab1*, *Pcolce2*, *Pde7b*, *Slc2a2*, *Socs1,* and *Tgm2* was altered in Pax5-overexpressing INS1 β cells (*n* = 8). (**F**) Enrichment of gene ontology terms among the genes with differential expression in Pax5-overexpressing INS1 β cells showed transcriptomic changes within pathways important for insulin secretion and cell numbers. *P < 0.05, **P < 0.01 ***P < 0.001, and ****P < 0.0001, by 2-tailed, paired *t* test (**A**–**E**). Box-and-whisker plots show the median, the 25th and 75th percentiles, and minimum and maximum values.

In view of the data shown in Figure 4J, we hypothesized that elevated Pax5 levels may affect cell numbers. This was supported by results from an MTT [3-(4,5-dimethylthiazol-2-yl)-2,5-diphenyltetrazolium bromide] assay, which measures metabolic activity and is used to investigate cell viability and numbers (Figure 6A). To investigate whether the reduced cell number is due to increased apoptosis and/or decreased proliferation, we quantified caspase-3/-7 activity and cleaved (i.e., active) caspase-3 as a measure of apoptotic activity, and used an 5-ethynyl-2′-deoxyuridine (EdU) incorporation assay to quantify proliferation. These analyses showed that Pax5 overexpression induced a clear increase in apoptotic activity and a drop in proliferation rates in INS1 β cells (Figure 6, B–D). Hence, we concluded that Pax5 is a regulator of β cell viability and numbers.

In a final effort to characterize the mechanisms underlying the profound effects of Pax5, we performed a global transcriptomic analysis of Pax5-overexpressing INS1 β cells. The analysis revealed that 3,069 genes were differentially expressed when comparing β cells overexpressing Pax5 and GFP, respectively (*q* < 0.05, Supplemental Table 9). These included 75 of the 395 T2D DEGs (Figure 6E and Supplemental Table 2, sheet A, and Supplemental Table 9). In addition to Pax5 itself, 3 of the other DEGs selected for functional follow-up, *Faim2*, *Pcolce2*, and *Slc2a2*, were altered in Pax5-overexpressing INS1 β cells, and in the same direction as in islets from individuals with T2D (Figure 6E). The 3,069 DEGs were enriched for many gene sets, including those for insulin secretion, glucose homeostasis, response to glucose, positive regulation of cell death, and aging (Supplemental Table 10 and Figure 6F). Overall, these data clearly demonstrated that Pax5 overexpression leads to transcriptomic changes that can have profound effects on β cell function and survival.

### PAX5 is a potential dysregulator of gene expression in T2D.

**Figure 7:**
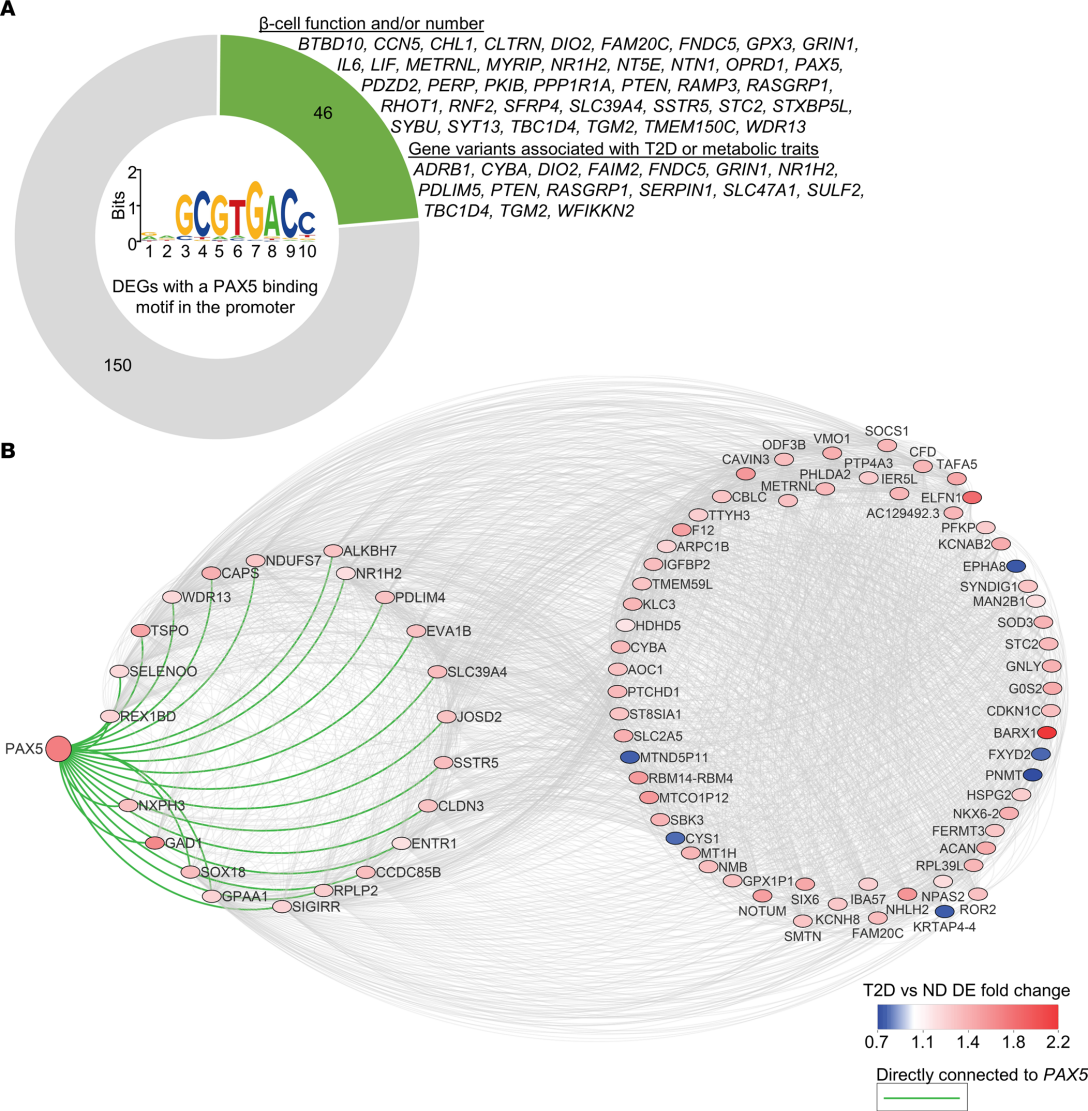
*PAX5* is potentially a key T2D DEG overexpressed in β cells. (**A**) Graphic showing the number of DEGs with a PAX5-binding motif in the promoter (based on a Pscan analysis) (30) and the proportion of these DEGs that have been shown to have a regulatory role in β cells or have genetic variants associated with T2D or metabolic traits in humans, either in this or other published studies. The PAX5-binding motif is shown in the center. (**B**) WGCNA (36) coexpression analysis based on weighted correlations among the 395 T2D DEGs showed that *PAX5* is part of an expression cluster containing 87 DEGs, with direct connection to 22 DEGs. DE, differential expression.

As PAX5 is a transcription factor, we next used the bioinformatics prediction tool Pscan (30) to test whether the 395 DEGs are enriched for genes with a PAX5-binding motif in the promoter. This would suggest that elevated PAX5 may cause dysregulation of other DEGs in T2D. Indeed, our analysis revealed that 196 DEGs had a putative PAX5-binding motif within the promoter, which is a significant enrichment (*P* = 0.017, Supplemental Table 11). Of note, the 196 genes included 6 of the genes we selected for functional follow-up (*BARX1*, *CHL1*, *FAIM2*, *HHATL*, *OPRD1*, and *PAX5* itself), and a literature search showed that approximately 25% of the 196 genes have been found to regulate β cell function and/or numbers, or have genetic variants associated with T2D or other metabolic traits in humans (Supplemental Table 11 and Figure 7A). These include *IL6* (31), *PPP1R1A* (32), *PTEN* (33), *SYT13* (34), and *TBC1D4* (35). Additionally, in human islets, the expression of *PAX5* correlated with 126 (32%) of the other 394 DEGs (Spearman’s correlation, *P* < 0.05, Supplemental Table 12), including *BARX1*, *CDKN1C*, *CHL1*, *ELFN1*, *GABRA2*, *GAD1*, *NEFL*, *PCOLCE2*, *PDE7B*, *SFRP1*, *SLC2A2*, *SOCS1*, *SYT1*, and *SYT12*. As a final piece of evidence supporting *PAX5* as a key DEG, we used weighted correlation network analysis (WGCNA) (36) for a coexpression analysis to establish whether *PAX5* is part of a dysregulated gene network in T2D. This analysis revealed a network with *PAX5* and 86 other T2D DEGs (Supplemental Table 13 and Figure 7B). Together, data from human islets and Pax5-overexpressing INS1 β cells suggest that PAX5 may cause dysregulation of many identified T2D-associated DEGs.

## Discussion

**Figure 8:**
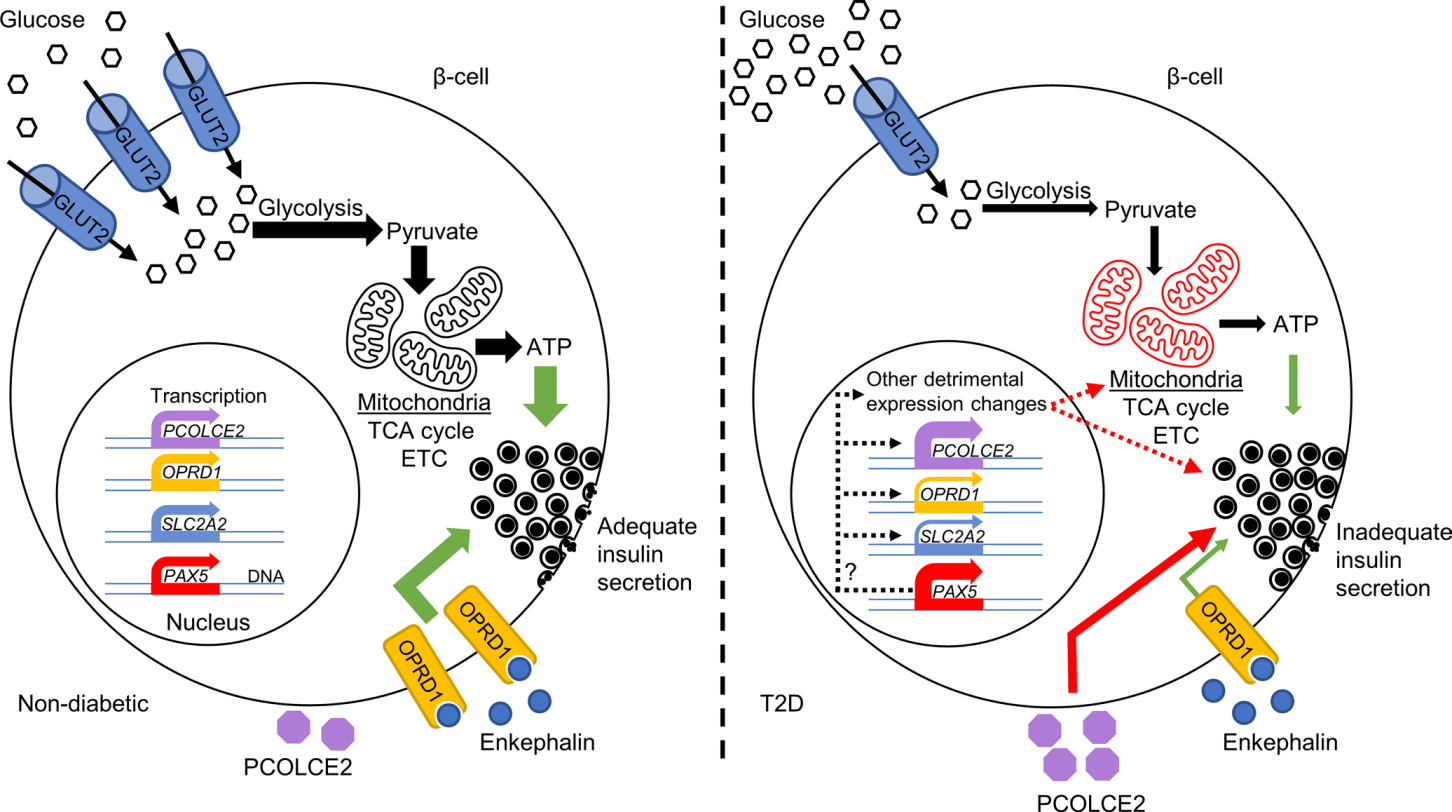
Schematic image presenting a model for how the T2D-associated changes may alter β cell function. Our analysis showed that the islet expression of 395 genes is altered in T2D. These expression changes are enriched for genes affecting, e.g., insulin secretion, and functional analyses showed that the T2D-associated changes to *OPRD1*, *PAX5*, *PCOLCE2*, and *SLC2A2* (encoding GLUT2) impair glucose-stimulated insulin secretion. In ND individuals, β cells highly express *SLC2A2*, leading to high levels of GLUT2 and efficient glucose uptake. This in turn, via glycolysis, the TCA cycle, and the electron transport chain (ETC) in well-functioning mitochondria, leads to ATP production, a higher cytosolic ATP/ADP ratio, and stimulation of insulin secretion. Simultaneously, our data suggest that signaling through OPRD1, an enkephalin receptor, stimulates insulin secretion. In T2D, there is dysregulation of *PAX5*, leading to greatly increased PAX5 mRNA and protein levels in β cells, as well as a severe reduction in *SLC2A2* and *OPRD1* expression. These changes lead to diminished insulin secretion, with PAX5 overexpression causing a strong reduction in mitochondrial activity. Simultaneously, *PCOLCE2* is upregulated, which, through an unknown mechanism, also impairs insulin secretion. Importantly, elevated *PAX5* may cause many of the other detrimental expression changes, including reduced *SLC2A2* expression. Green arrows indicate stimulation, red arrows indicate inhibition, and dashed arrows indicate potential effects.

This study identified T2D candidate genes with altered expression in pancreatic islets from individuals with T2D versus islets from ND controls. Their altered expression may be a contributing factor in the development of T2D, as many identified DEGs associated with HbA1c levels in a cohort of islets from individuals not previously diagnosed with T2D. Many DEGs displayed alterations in chromatin state or DNA methylation in T2D islets, and SNPs associated with DEGs affected T2D and metabolic traits. Rodent in vivo data also supported the importance of our findings, as mouse strains deficient for identified DEGs had altered glucose homeostasis and body composition. Functional validation of top-ranked DEGs clearly supported a role for the identified expression changes in T2D, as mimicking the changes for *OPRD1*, *PAX5*, and *SLC2A2* in human islets and clonal β cells perturbed insulin secretion. Extensive characterization of the Pax5-induced defect showed that impaired mitochondrial activity is a likely culprit. Our data further supported *PAX5* as a key T2D DEG that drives the change in expression of other DEGs. On the basis of the present findings, we propose the model presented in Figure 8, in which increased PAX5 levels contribute to impaired insulin secretion through mitochondrial dysfunction and transcriptional regulation of other genes, e.g., *SLC2A2* and *OPRD1*, in human islets.

Most of the identified DEGs, including *PAX5*, *OPRD1*, *PCOLCE2*, *CDKN1C*, *GABRA2*, *IL6*, *PDE7B*, *SIRT1*, *SOCS1*, *SYT1*, *SYT12*, *SYT14*, and *TET1*, represent findings not observed in previous transcriptomic analyses of T2D islets (1–8). However, we also replicated nearly 100 known T2D DEGs, including *SLC2A2*, *CHL1*, *GLRA1*, *IAPP*, *PPP1R1A*, *PTEN*, and *SYT13* (1–8), supporting a robust data set. The number of identified DEGs overlapping with genes identified in more than one of the published studies was low, possibly due to limited sample sizes in the previous studies or varying pathophysiological and demographic profiles between individual donors, as well as to differences in islet isolation and culturing protocols, analysis platforms, and variables corrected for in the statistical analysis. For example, while we used RNA-Seq, Solimena et al. primarily used microarray to analyze the transcriptome (6). The overlap was even smaller when comparing with scRNA-Seq analyses, potentially explained by the small number of islet donors in single-cell studies (*n* = 5–12 ND donors; *n* = 3–6 donors with T2D) and/or a greater number of variables adjusted for in our study [(age + sex + islet purity + DIC) versus single-cell studies (sex + ethnicity)] (3, 5, 8, 11). It should also be noted that the DEGs described by the published single-cell studies largely do not overlap (10), potentially because of different criteria for expression filtering, use of different statistical and bioinformatics tools, and different numbers of cells studied (638 [ref. 3], 1,492 [ref. 8], 2,209 [ref. 5]). Importantly, the DEGs identified in our analysis were enriched for gene ontology terms important for β cell function including hormone secretion, divalent inorganic cation homeostasis, and growth factor binding. Our functional validation also showed that the expression changes may contribute to T2D pathophysiology, as knockdown of *SLC2A2* or *OPRD1* and overexpression of Pax5 resulted in impaired GSIS. Additionally, the expression of many DEGs, including *OPRD1*, *PAX5*, and *SLC2A2*, associated with HbA1c in ND individuals and individuals not diagnosed with T2D, with the direction of association indicating that the changes may predispose to disease.

PAX5, a transcription factor important for B lymphocyte development and frequently mutated in acute B lymphoblastic leukemia (37), is barely expressed in human islets from ND individuals (38). While low islet PAX5 expression in ND individuals was confirmed in our cohort, expression at both mRNA and protein levels was upregulated in T2D, with immunostaining showing that the upregulation took place in β cells. Interestingly, T2D and SNPs were associated with altered DNA methylation of *PAX5* in human islets, suggesting potential epigenetic regulation. PAX5, which can act as both an activator and repressor (39), belongs to a transcription factor family with several members. Notably, PAX2, -4, and -6, are important for pancreas development and islet function (40). In our analysis, *PAX6* was highly expressed in human islets, while *PAX2* and *PAX4* were expressed at low levels. Although there were no differences in the expression of *PAX2* or *PAX6*, the expression of *PAX4* was nominally reduced in islets from individuals with T2D versus those from ND controls (*P* = 0.004). We showed that PAX5 upregulation was associated with reduced mitochondrial function, perturbed GSIS, and β cell loss. Interestingly, our analyses showed that *PAX5* may be a key T2D DEG causing dysregulation of many other DEGs. This was supported by our analysis of Pax5-overexpressing INS1 β cells, which exhibited vast transcriptomic changes, including for genes selected for functional follow-up (*Faim2*, *Pcolce2*, and *Slc2a2*) and for many other T2D DEGs, altered in the same direction in this model as in islets from individuals with T2D. Mice heterozygous for *Pax5* KO exhibit impaired glucose tolerance, indicating that Pax5 deficiency somehow exerts negative effects on glucose homeostasis in peripheral tissues. Data from the IMPC (www.IMPC.org) show that heterozygous *Pax5* animals exhibit changes in white blood cell numbers, which could cause a cytokine imbalance and a resulting insulin resistance peripherally, as seen in SCID mice completely lacking mature B and T lymphocytes (41). It should be noted that the Pax5 overexpression in INS1 β cells may be greater than in islets of individuals with T2D, and it is therefore uncertain how strong the effects of elevated PAX5 are in T2D. The proteins overexpressed in our study were HA tagged, and it is possible that the tag contributed to the defect in overexpressing cells. For Pax5, however, this is unlikely, as overexpression of the untagged protein perturbed GSIS in a manner similar to that by the tagged protein .

*SCL2A2* encodes GLUT2, the main glucose transporter in rodent β cells, as evidenced by the impaired glucose tolerance in *Slc2a2*-KO mice (42). Its role in human islets has been questioned (43), but *SLC2A2* mutations cause transient neonatal diabetes (28). Our data indicated that GLUT2 plays an important role also in adult human β cells, as its knockdown in human islets impaired insulin secretion. In INS1 β cells, Pax5 overexpression downregulated *Slc2a2*, indicating that reduced Glut2-mediated glucose uptake may contribute to the perturbed mitochondrial function and secretory defect observed in this model. We also found that *PAX5* correlated negatively with *SLC2A2* expression in islets from the LUDC case-control cohort, and PAX5 may hence directly downregulate *SLC2A2* also in human islets. *OPRD1* encodes a receptor for enkephalins, which have been shown to both inhibit and stimulate insulin secretion (44–47) through potential dose-dependent effects (45). Enkephalin is expressed in a small subset of islet cells (38) and may regulate insulin secretion via paracrine signaling. While *OPRD1* knockdown reduced GSIS in human islets by only approximately 20%, it should be remembered that T2D is a disease characterized by dysregulation of many genes that contribute to a varying extent to cellular dysfunction. The protein encoded by *PCOLCE2* binds to procollagens (48). Increased *PCOLCE2* expression, as occurs in T2D islets, perturbed insulin secretion, but the mechanism remains unknown. *CHL1* encodes an adhesion protein, and its islet expression has been shown to be reduced in T2D (7). *Chl1* knockdown in rodent β cells lines, including INS1 β cells (26), has also been shown to impair GSIS. In our hands, *Chl1* was not detected in INS1 β cells (data not shown), and *CHL1* knockdown in human islets only reduced insulin secretion significantly at basal glucose levels. The importance of reduced *CHL1* expression in T2D therefore remains unclear. As with *SLC2A2*, we present evidence supporting the idea that PAX5 may regulate *CHL1*, *OPRD1*, and *PCOLCE2*. While manipulation of some of the genes we selected for functional follow-up did not alter cellular function, they may still play roles in islets in vivo or in T2D.

Many of the identified DEGs we did not functionally validate are highly relevant to T2D. For example, *GLRA1* belongs to the DEGs already reported on by others (7) and has lower expression levels in β cells from individuals with T2D (8). It encodes a glycine receptor, and silencing of *Glra1* in clonal β cells reduces GSIS (49). Of note, a 48-hour treatment of ND human islets with high glucose, palmitate, or high glucose plus palmitate increased DNA methylation and decreased the expression of *GLRA1* (49–51). Here, we found an inverse correlation between HbA1c levels and *GLRA1* expression in the LUDC islet HbA1c cohort. These data support the notion that reduced *GLRA1* expression in β cells, potentially due to epigenetic alterations, may predispose individuals to T2D. In agreement with other studies (1, 5, 6), we found reduced *IAPP* expression in islets from individuals with T2D. *IAPP* encodes islet amyloid polypeptide, or amylin, which is costored and cosecreted with insulin. In T2D, it forms amyloid depots in islets and is associated with cell death and pancreatic dysfunction (52). Moreover, we show that *TET1* was downregulated in islets from individuals with T2D. In line with this, we previously found reduced *TET1* expression in adipose tissue from individuals with T2D versus tissue from ND controls (53, 54). TET1 oxidizes DNA methylation to hydroxymethylation and is known to be important for β cell differentiation (55). Additionally, we identified 4 synaptotagmin-encoding DEGs — *SYT1*, *SYT12*, *SYT13*, and *SYT14*, of which *SYT13* was identified by earlier studies and is differentially expressed in β cells from individuals with T2D (3, 6, 34). As synaptotagmins are involved in Ca^2+^-triggered exocytosis (56), their differential expression may contribute to altered exocytosis in T2D islets. Indeed, synaptotagmins 1 and 13 have been shown to affect insulin secretion (34, 57, 58). *CDKN1C*, an upregulated DEG, encodes a cell-cycle inhibitor, and demethylation of the *CDKN1C* promoter in human islets results in β cell proliferation (59). *PDE7B* encodes a cAMP phosphodiesterase, which we found upregulated in T2D islets, and we previously showed that upregulation of this gene reduces GSIS (60). Many other DEGs, e.g., *PTEN*, *PPP1R1A*, *IL6*, *SIRT1*, and *SOCS1*, have also been shown to affect β cell function or numbers (31–33, 61, 62). Of note, *PPP1R1A* is differentially expressed in β cells from individuals with T2D (8).

Moreover, some of our DEGs identified in islets have previously been found to be differentially expressed also in other tissues from individuals with T2D versus ND control tissues. For example, *C1S*, *CTSZ*, *PTGES*, *SCYL2*, *SERPING1*, and *TET1* are differentially expressed in adipose tissue (53), whereas *SLC39A4*, *MNS1*, *RAB33B*, and *ACP2* are differentially expressed in muscle cells (63), and *PPP1R1A* is differentially expressed in liver (64). Stable epigenetic modifications taking place during embryogenesis, mQTLs, eQTLs, or ectopic fat accumulation may contribute to similar gene regulation in multiple tissues.

This study has some potential limitations. For example, we analyzed whole islets, which include several cell types. Hence, to understand which cell types expressed T2D-associated DEGs, we studied sorted human α and β cells as well as scRNA-Seq data from human islets. This approach showed that 93% of the identified DEGs were expressed in β cells. IHC analysis also showed that our main candidate, PAX5, was upregulated in human β cells. Moreover, the bulk RNA-Seq approach allowed us to analyze islets from a large cohort, as well as much larger numbers of cells from each islet preparation than the scRNA-Seq approach would. Importantly, we replicated some DEGs identified in other islet T2D case-control cohorts, where either single-cell or bulk RNA-Seq was used. It was beyond the scope of this study to follow up identified DEGs in all islet cell types, and future studies should investigate gene manipulations in cultured α, δ, and PP cells. For instance, *IGFBP2*, *NPAS2*, *NDUFS7*, and *TGM2* showed differential expression in α cells from individuals with T2D (3, 5, 8) and have previously been shown to affect diabetes and islet-related phenotypes or mitochondrial function (65–68). Even though our islet cohort is among the most extensive of its kind, it would be beneficial to analyze islets from an even larger number of donors and study whether racial and ethnic background impact islet gene expression in individuals with T2D. Unfortunately, islet supply is limited, and we had to perform the overexpression experiments in clonal β cells. The investigated cells were thus not in their normal 3D structure, surrounded by the other islet cell types, which may have influenced the results. Moreover, HbA1c levels may fluctuate because of, e.g., medication and red blood cell count. A minor subset of individuals defined as ND in our cohort, based on an HbA1c of less than 42 mmol/mol, may hence be prediabetic. Finally, although the IMPC in vivo data support a role for the identified DEGs in glucose homeostasis, KO strains for many genes are lacking, and given the available data, it is difficult to establish whether metabolic defects are due to islet and/or peripheral effects.

In conclusion, by studying one of the largest existing T2D islet case-control cohorts, we have identified differentially expressed T2D candidate genes. Collectively, our in vivo and in vitro analyses clearly support a role for these gene expression changes in the dysregulation of insulin secretion and, thereby, in T2D pathophysiology. We demonstrated that *PAX5* expression was induced in β cells in T2D and that this could underlie transcriptional changes to many other T2D-associated DEGs. Our data further showed that elevated PAX5 levels resulted in reduced glucose-induced mitochondrial activity and, consequently, a reduced cytosolic ATP/ADP ratio and perturbed insulin secretion. Given the strong effects on β cell function, we propose that the changes in gene expression we identified could contribute to T2D pathophysiology and that the DEGs thus could be targeted in novel strategies for T2D treatment.

## Methods

Full details can be found in the Supplemental Methods.

### Human islets.

Human islets of Langerhans were obtained from the Human Tissue Laboratory, which is funded by the Excellence of Diabetes Research in Sweden (EXODIAB) network (www.exodiab.se/home) in collaboration with the Nordic Network for Clinical Islet Transplantation Program (www.nordicislets.org). Islets from cadaver donors were prepared by enzymatic digestion and density gradient separation. Islet preparation purity and counts were determined as described previously (69). Details on islet purity, measured by dithizone staining of individual islet preparations, are presented in Supplemental Table 14.

### RNA-Seq.

RNA was extracted from human pancreatic islets using the AllPrep DNA/RNA kit or the miRNeasy Mini Kit (QIAGEN). Sample preparation of 1 μg high-quality RNA was done using the TruSeq RNA Library Preparation Kit or the TruSeq Stranded Total RNA Library Prep kit, followed by RNA-Seq on a HiSeq 2000 or NextSeq 500 (Illumina), respectively.

### Data and material availability.

All data needed to evaluate the conclusions in this study are presented here and/or in the Supplemental Methods. The human islet RNA-Seq data sets were deposited in the LUDC repository (https://www.ludc.lu.se/resources/repository) under the following accession numbers: LUDC islet case-control cohort (accession no. LUDC2022.07.111), LUDC sorted α/β cell cohort (accession no. LUDC2022.07.112), and LUDC islet HbA1c cohort (accession no. LUDC2022.07.113). Data have also been deposited at the European Genome-Phenome Archive (https://www.ebi.ac.uk/ega/) under accession number EGAD00001005512. Individual-level sequencing and clinical data from the human pancreatic islet donors are not publicly available due to ethical and legal restrictions related to the Swedish Biobanks in Medical Care Act, the Personal Data Act, and European Union’s General Data Protection Regulation and Data Protection Act. The ATAC-Seq data set and mRNA expression data from GFP- and Pax5-overexpressing INS1 β cells are available in the NCBI’s Gene Expression Omnibus (GEO) repository under accession numbers GSE129383 and GSE211310, respectively.

### Statistics.

The functional data were analyzed with a 2-tailed, paired *t* test unless stated otherwise. Other analyses were performed as described in the respective sections in the Supplemental Methods. Data are presented as the mean ± SEM, unless stated otherwise. A *P* or *q* value (when correction for multiple testing was required) below 0.05 was considered statistically significant.

### Study approval.

Written informed consent was obtained from pancreatic donors or their relatives, and all procedures were approved by the Swedish Ethical Review Authority (Permit number 2011263).

## Author contributions

KB, AP, AK, EC, JKO, LBB, TR, AL, C Luan, SR, MN, ÅN, SG, CLL, JOL, MB, JLSE, PV, MF, HM, NW, UK, IA, LE, RBP, LRC, and C Ling contributed to the study design and read and edited the manuscript. KB, AP, AK, EC, JKO, LBB, TR, AL, C Luan, SR, MN, ÅN, SG, CLL, PV, NW, UK, RBP, LRC, and C Ling performed experiments and data analysis. KB and C Ling wrote the original draft of the manuscript.

## Supplementary Material

Supplemental data

Supplemental table 2

Supplemental table 3

Supplemental table 4

Supplemental table 5

Supplemental table 6

Supplemental table 7

Supplemental table 8

Supplemental table 9

Supplemental table 10

Supplemental table 11

Supplemental table 12

Supplemental table 13

Supplemental table 14

## Figures and Tables

**Table 1:**
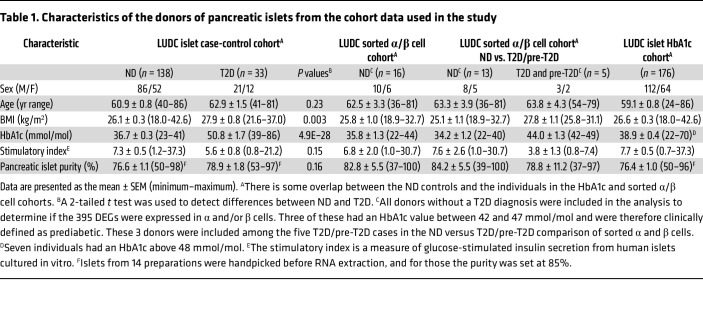
Characteristics of the donors of pancreatic islets from the cohort data used in the study

## Version 1 01/19/2023

In-Press Preview

## Version 2 02/15/2023

Electronic publication

## Version 3 12/21/2023

Figure 4D-E corrected to one star for the statistical comparisons

## Version 4 04/15/2026

Corrected text in the Data and material availability section
