# Corrigendum to Type 2 diabetes candidate genes, including PAX5, cause impaired insulin secretion in human pancreatic islets

**DOI:** 10.1172/JCI206633

**Published:** 2026-04-15

**Authors:** Karl Bacos, Alexander Perfilyev, Alexandros Karagiannopoulos, Elaine Cowan, Jones K. Ofori, Ludivine Bertonnier-Brouty, Tina Rönn, Andreas Lindqvist, Cheng Luan, Sabrina Ruhrmann, Mtakai Ngara, Åsa Nilsson, Sevda Gheibi, Claire L. Lyons, Jens O. Lagerstedt, Mohammad Barghouth, Jonathan L.S. Esguerra, Petr Volkov, Malin Fex, Hindrik Mulder, Nils Wierup, Ulrika Krus, Isabella Artner, Lena Eliasson, Rashmi B. Prasad, Luis Rodrigo Cataldo, Charlotte Ling

Original citation: *J Clin Invest*. 2023;133(4):e163612. https://doi.org/10.1172/JCI163612

Citation for this corrigendum: *J Clin Invest*. 2026;136(8):e206633. https://doi.org/10.1172/JCI206633

After publication, the authors became aware of incorrect information in the *Data and material availability* section. The corrected text has been added and the HTML and PDF versions of the paper have been updated.

The authors regret the error.

## Version 1 04/15/2026

Electronic publication

