# Supplementary material for: Type 2 diabetes candidate genes, including PAX5, cause impaired insulin secretion in human pancreatic islets
Source: J Clin Invest. 2023 Jan 19;133(4):e163612. doi: 10.1172/JCI163612 (PMC9927941; doi:10.1172/JCI163612)
Supplement: Supplemental data [file jci-133-163612-s010.pdf]

Type 2 diabetes candidates in human pancreatic islets, including PAX5, causing impaired insulin secretion – Supplementary material

Karl Bacos,[1]* Alexander Perfilyev,[1]† Alexandros Karagiannopoulos,[2]† Elaine Cowan,[2]† Jones K. Ofori,[1]† Ludivine Bertonnier-Brouty,[3] Tina Rönn,[1] Andreas Lindqvist,[4] Cheng Luan,[5] Sabrina Ruhrmann,[1] Mtakai Ngara,[4] Åsa Nilsson,[6] Sevda Gheibi,[7] Claire L. Lyons,[7] Jens O. Lagerstedt,[2]‡ Mohammad Barghouth,[5] Jonathan L.S. Esguerra,[2]‡ Petr Volkov,[1] Malin Fex,[7] Hindrik Mulder,[7] Nils Wierup,[4] Ulrika Krus,[6]‡ Isabella Artner,[3] Lena Eliasson,[2] Rashmi Prasad,[8#] Luis Rodrigo Cataldo,[7,9#] and Charlotte Ling[1]*

[1]Epigenetics and Diabetes Unit, Department of Clinical Sciences, Lund University Diabetes Centre, Scania University Hospital, Malmö, Sweden.
[2]Unit of Islet Cell Exocytosis, Department of Clinical Sciences, Lund University Diabetes Centre, Scania University Hospital, Malmö, Sweden.
[3]Endocrine Cell Differentiation, Department of Laboratory Medicine, Lund Stem Cell Center, Malmö, Sweden.
[4]Neuroendocrine Cell Biology, Department of Clinical Sciences, Lund University Diabetes Centre, Malmö, Sweden.
[5]Unit of Islet Pathophysiology, Department of Clinical Sciences, Lund University Diabetes Centre, Malmö, Sweden.
[6]Human Tissue Lab, Department of Clinical Sciences, Lund University Diabetes Centre, Scania University Hospital, Malmö, Sweden.
[7]Molecular Metabolism Unit, Department of Clinical Sciences, Lund University Diabetes Centre, Scania University Hospital, Malmö, Sweden.
[8]Genomics, Diabetes and Endocrinology, Department of Clinical Sciences, Lund University Diabetes Centre, Scania University Hospital, Malmö, Sweden.
[9]The Novo Nordisk Foundation Centre for Basic Metabolic Research, Faculty of Health and Medical Sciences, University of Copenhagen, Copenhagen, DK-2200 Denmark.

†Equal contribution
[#]Equal contribution
‡Current affiliation: Novo Nordisk A/S, Copenhagen, Denmark

Content:

Supplementary methods

Table S1

Figure S1-5

Supplementary Methods

RNA-sequencing

RNA was extracted from human pancreatic islets using AllPrep DNA/RNA kit or miRNeasy Mini Kit (Qiagen, Hilden, Germany). Sample preparation of 1 μg high quality RNA was done using TruSeq RNA Library Preparation Kit or TruSeq Stranded Total RNA Library Prep, followed by RNA-sequencing on HiSeq 2000 or NextSeq 500 (Illumina, San Diego, CA, USA), respectively. For preparation of data and analysis, quality and adapter trimming was done using Trim Galore (https://www.bioinformatics.babraham.ac.uk/projects/trim_galore/), and then Salmon (1) was used for quantification of transcript expression. Quality control (QC) was done using fastQC (https://www.bioinformatics.babraham.ac.uk/projects/fastqc/) and multiQC (2) tools. Downstream analysis was done using R (http://www.R-project.org/). R package tximport (3) was used to import transcript-level abundance, estimated counts and transcript lengths into R using gencode annotation v. 32. Genes with ≥3 reads in ≥10% of samples were considered expressed. The criteria for inclusion of islet preparations in further analysis were islet purity >50% and days in culture ≤7 days (all preparations), as well as >40 years of age and no previous gestational diabetes (preparations from ND controls). Differential expression analysis and identification of DEGs was based on a generalized linear model using DESeq2 (4), with correction for age, sex, islet purity, and days in culture. We performed an additional analysis where we also corrected the generalized linear model for BMI. Deseq2 was also used to generate normalized gene expression counts. The analysis of association between HbA1c and gene expression was performed as above on data from all 176 islet preparations from individuals without a clinical T2D diagnosis for which we had HbA1c values, with the exclusion of an individual with previous gestational diabetes. The analysis was corrected for age, sex, BMI, islet purity, and days in culture.

Overlap between our DEGs and differentially expressed genes in published studies

Public data on differentially expressed genes in islets from ND controls versus individuals with T2D were integrated into lists in the following way; Ensembl IDs were preferred over gene symbols/names due to their more consistent annotation. Ensembl IDs were used directly when available, otherwise, gene symbols were matched to their Ensembl IDs using the R package biomaRt (GRCh38 - Ensembl v.100). Genes that were not assigned to an ID were scanned for synonyms/aliases with biomaRt and a second round of Ensembl ID matching was performed. Unmatched genes without Ensembl IDs were removed from subsequent analyses. Genes with multiple Ensembl IDs were represented by their first matched Ensembl ID.

Pathway analysis

The R package WebGestaltR (v.0.4.4) was used to discover enriched functional terms and pathways in our datasets. Over-representation analysis using the human genome as reference was performed against Gene Ontology (Release 2021-11-16) with an enrichment threshold of FDR below 5% ($q<0.05$).

Gene expression in sorted α- and β-cells

Pancreatic islets from 18 donors (13 ND, 3 pre-T2D based on HbA1c 42-48 mmol/mol, and 2 T2D, see Table 1 in the main text) were dispersed, fixed and stained as previously described (5), with the exception that MAB1249 (R&D Systems, Minneapolis, MN, USA) was used for glucagon staining. The sorting was performed on an Aria Fusion (BD Biosciences, San Jose, CA, USA). RNA was isolated from sorted cells with the RecoverAll Total Nucleic Acid Isolation Kit (ThermoFisher Scientific, Waltham, MA, USA). Libraries for sequencing were prepared with the TruSeq Stranded Total RNA Library Prep kit (Illumina) and sequencing was done on a NextSeq 500 (Illumina). The data were analyzed as described above, with adjustment for individual id as covariate in DESeq2 (4).

eQTL analysis

Genome-wide genotyping for islet samples in the *LUDC islet case-control cohort* (134 ND and 33 donors with T2D) was run on the OmniExpress genotype arrays on the Illumina platform. Quality of genotyping was assessed before imputation separately and samples were excluded if they had an overlap genotype success rate <95%. Quality control was performed using the PLINK1.9 software (6). Briefly, samples with missingness rate of genotype data larger than 5% were removed and we assessed concordance between reported sex and the sex based on genotype data. Loci with minor allele count (MAC) smaller than five or a p-value smaller than $1x10^{-4}$ were removed. Heterozygosity of the samples was checked and none of the samples were outside three standard deviations from the mean. Finally, no duplicate or related samples were found, and monomorphic sites were removed. Michigan imputation server (https://imputationserver.sph.umich.edu/) was used for imputation utilizing HRC version r1.1 as a reference panel. Imputation was conducted using the Sanger imputation service (https://imputation.sanger.ac.uk/) using MINIMAC3 and Haplotype Reference Consortium version r1.1 as a reference panel with array build hg38. SNPs with an INFO score >0.4 and Hardy–Weinberg Equilibrium $p>1x10^{\wedge -6}$ (for chrX this was calculated from female individuals only) from each panel were kept. A vcf for each chromosome was generated. Dosages were calculated from the imputation probabilities and converted into best-guess genotypes using PLINK. Files from all chromosomes were merged and transposed.

The RNA-sequencing data of our 395 DEGs from the *LUDC islet case-control cohort* were normalized for sequencing depth for the selected genes and further processed by quantile normalization to obtain normal distribution while preserving the ranks. A *cis*-eQTL analysis was implemented on the Matrix eQTL software using a linear model with age, sex, BMI, days in culture, islet purity and T2D status as covariates. Association of genotype with gene expression of our 395 DEGs was computed within a window of SNPs located 500kb upstream and 500kb downstream of respective gene, and effects are presented as beta coefficients and t-statistics. Significance levels are indicated by raw p-values and multiple testing correction by FDR adjustment. A subset of the donors included in the LUDC eQTL analysis were also included in the INSPIRE eQTL analysis.

Identification of SNPs associated with T2D and/or glycemic traits

We downloaded SNPs associated with T2D and/or glycemic traits (corrected insulin response (CIR), Disposition index (DI), HOMA-B, fasting glucose (FG), fasting insulin (FI), HbA1c) based on genome-wide significance (p-value $\leq 5x10^{-8}$) from the Common Metabolic Diseases (CMD) Knowledge Portal (https://hugeamp.org). In this analysis, we investigated SNPs that

reside in regions stretching from 500kb upstream of the transcription start site to 500kb downstream of the transcription end site of the 395 DEGs.

Mouse models

The International Mouse Phenotyping Consortium (IMPC) is a joint initiative between 19 research organizations across Europe, North America and Asia, whose goal is to generate a comprehensive, robust, reproducible, and freely accessible catalogue of functions for all mammalian genes. The IMPC uses standardized operating procedures, to create and phenotype global knock out strains for individual genes, which ensures the quality of its data and makes it an excellent resource for the global scientific community (7, 8). Where possible, we retrieved and collated IMPC data for three relevant metabolic based phenotypic measurements (data extracted on April 15th, 2020). These were: 1. insulin blood level (IB), 2. intraperitoneal glucose tolerance test (IPGTT) with readings for three parameters; area under glucose response curve (AUCG), initial response to glucose challenge (IGR) and fasted blood glucose concentration (FG) and, 3. DEXA (dual-energy X-ray absorptiometry) analysis with readings for two parameters; fat and lean mass adjusted by total body weight (Fat/BW and Lean/BW). All IMPC data retrieved for our analysis were generated from experiments performed in single-gene adult KO (7M/7F) and wild type (large variable n) mice on a C57BL/6N background (11-16 weeks), and for the purposes of our study we considered a p-value <0.05 significant.

siRNA transfection of human islets

100-200 islets were seeded in culture dishes containing RPMI media with 5mM glucose, 10% FBS, 200mM L-glutamine. A final transfection volume of 2.5mL per dish contained 50nM of Silencer® Select Pre-Designed siRNA against *OPRD1* (siRNA ID s9862), *CHL1* (s21124), *HHATL* (s33086) or *SLC2A2* (s12928) in Opti-MEM reduced serum media and 6.25µL of Lipofectamine RNAiMAX (all from ThermoFisher Scientific). A second transfection was performed 24h after the first transfection and all functional experiments were performed 72h after the first transfection. In total, islets from eight donors were transfected.

RNA extraction and quantification by Real-time Quantitative PCR

Total RNA was extracted from siRNA-transfected human islets with the miRNeasy isolation kit (QIAGEN) and converted to cDNA with the RevertAid First Strand cDNA synthesis kit (ThermoFisher Scientific). Quantitative PCR (qPCR) was performed in triplicates on a 384-well plate using Applied Biosystems QuantStudio 7 Flex Real Time PCR system (ThermoFisher Scientific) under default cycling parameters. TaqMan assays were used to measure mRNA expression of *OPRD1* (assay ID Hs00538331_m1), *CHL1* (Hs00544069_m1), *HHATL* (Hs00375325_m1), and *SLC2A2* (Hs01096908_m1). We used *PPIA* (Hs04194521_s1) and *HPRT1* (Hs02800695_m1) to normalize the mRNA expression. All TaqMan assays and qPCR reagents were purchased from ThermoFisher Scientific. Threshold levels of all Ct values were automatically set, and mRNA expression was normalized using the geometric means of the two endogenous controls. Relative expression was calculated with the ΔΔCt method.

Insulin secretion assay (human islets)

Batches of 10-12 human islets were preincubated for 30 minutes in KREBS buffer (2.5mM CaCl2, 4.7mM KCl, 120mM NaCl, 25mM $NaHCO_3$, 1.2mM KH_2PO_4, 1.2mM $MgSO_4$, and 10mM HEPES) with 2.8mM glucose before stimulation for 1h in KREBS with either 2.8mM or 16.7mM glucose. After stimulation, islets were dissolved in 100μL RIPA buffer (50mM TRIS-HCl,150mM NaCl, 0.5mM NaDeoxycholate, 2mM EDTA, 50mM NaF, 1% Triton-X, 0.1% SDS). Insulin was measured by ELISA (#10-1113-01, Mercodia, Uppsala, Sweden) and secreted insulin normalized to the total insulin content.

Cell culture, transfection and transduction

The clonal rat β-cell line 832/13 INS1 (9), a kind gift from Professor Christopher Newgard (Duke University, Durham, NC, USA), was used for overexpression experiments. C-terminally HA-tagged (a three amino acid glycine linker followed by the tag) cDNA sequences for *Barx1*, *Elfn1*, *Faim2*, *Nefl*, *Pax5*, *Pcocle2*, and *Sfrp1* were cloned into the pcDNA3.1 expression vector by GenScript (Piscataway, NJ, USA). The plasmids were transfected into INS1 β-cells as previously described (10). A plasmid containing the HA-tagged cDNA of GFP was used as control. Lentiviruses containing HA-tagged (as above) cDNA of *Barx1*, *Nefl*, *Pax5*, *Pcolce2*, or *Sfrp1*, upstream of an internal ribosomal entry site and the cDNA for GFP, were produced by Lund University Cell and Gene Therapy core facility. A lentivirus containing only the HA-tagged cDNA of GFP was used as control. Expression of the respective cDNA was driven by the human phosphoglycerate kinase 1 promoter. Cells were transduced with 1.25 viral particles per cell for 24h and all experiments performed 72h after initiation of transduction.

Insulin secretion in INS1 β-cells

Transduced INS1 β-cells were washed in secretion assay buffer (SAB; 114mM NaCl, 4.7mM KCl, 1.2mM KH_2PO4, 1.16mM $MgSO_4$, 20mM HEPES, 2.5mM $CaCl_2$, 25.5mM $NaHCO_3$, 0.2% BSA, pH 7.2) supplemented with 2.8mM glucose, followed by a 2h pre-incubation at 37 °C in the same buffer. Insulin secretion was then determined by stimulating the cells with fresh SAB with 2.8 or 16.7mM glucose for 1h. For secretion experiments with high K^+, NaCl was proportionally decreased to maintain osmolarity in the buffer. After stimulation, cells were dissolved in RIPA buffer. Insulin was measured by ELISA (#10-1145-01, Mercodia) and secreted insulin normalized to the total insulin content. Total protein in each well was measured with the BCA Protein Assay Kit (ThermoFisher Scientific).

Immunohistochemistry

PAX5 expression was examined in human pancreas sections from five ND controls and four individuals with T2D. Antibodies against PAX5 (ab109443, (Abcam, Cambridge, UK), 1:250 dilution) and insulin (A0564, (Agilent, Santa Clara, CA, USA), 1:1000 dilution) were used. Immunohistochemistry was performed as previously described (11). Briefly, sections were incubated overnight at 4ºC, followed by two 10-minute washes in PBS with 0.25% Triton X-100. Thereafter slides were incubated for 60 minutes at room temperature with secondary antibody (Jackson ImmunoResearch, West Grove, PA, USA); donkey anti-rabbit Cy2 (711-225-152, 1:400 dilution) and donkey anti-guinea pig Alexa Fluor594 (706-585-148, 1:400 dilution). Nuclei were counter-stained with DAPI. After incubation and two 10-minute washes

in PBS with 0.25% Triton X-100, cover slides were mounted in PBS:glycerol (1:1). Immunofluorescence was examined in an epi-fluorescence microscope (Olympus BX60, Olympus, Tokyo, Japan). Images were taken with a digital camera (DP74, Olympus) using the CellSens software (Olympus). All antibodies were diluted in PBS with 0.25% Triton X-100 and 0.25% BSA.

Mitochondrial oxygen consumption measurements

Mitochondrial oxygen consumption rate (OCR) was evaluated with an XFe24 extracellular flux analyzer (Agilent). Transduced INS1 β-cells were starved in SAB containing 2.8mM glucose for 2h and OCR was measured in unbuffered SAB (i.e., without bicarbonate and HEPES) every 3 minutes for 90 minutes. OCR was measured at basal glucose (2.8mM glucose) and after addition of 16.7mM glucose, 5μM oligomycin, 4μM Carbonyl cyanide p-trifluoromethoxyphenylhydrazone (FCCP), and 1μM Rotenone/Antimycin A. Wave Seahorse Software and Seahorse Analytics online tool (seahorseanalytics.agilent.com) were used to analyze the data. Non-mitochondrial respiration was subtracted, and OCR data normalized to total protein content as measured by BCA assay.

ATP/ADP ratio

The ATP/ADP ratio was measured with the fluorescent biosensor PercevalHR. Approximately 70,000 cells were seeded on poly-D-lysine (1 mg/mL) coated Lab-Tek chambered cover glass (ThermoFisher Scientific). INS1 β-cells were transfected with Lipofectamine 3000 (ThermoFisher Scientific) and 1μg of pcDNA3.1 or pcDNA3.1-Pax5, together with 1μg of PercevalHR plasmid DNA (Addgene ID: #21737) per well. 72h later, the cells were pre-incubated in experimental buffer (pH 7.4): 3.6mM KCl, 1.3mM $CaCl_2$, 0.5mM $MgSO_4$, 0.5mM Na_2HPO_4, 10mM HEPES, 5mM $NaHCO_3$ and 135mM NaCl, supplemented with 2.8mM glucose, for 90 minutes at 37°C. Cover glass with adhered cells were mounted on the stage of a Zeiss Axiovert 200 M microscope (Carl Zeiss AB, Stockholm, Sweden) equipped with confocal unit. PercevalHR was excited with laser light at 488 nm and emission was detected at 520 nm.

Western blot analysis in INS1 β-cells

Total protein was extracted and separated by 4–15% TGX Stain-Free gels (Bio-Rad, Hercules, CA, USA). The gels were then activated with ultraviolet light for 1 minute to visualize total protein on the blotted membrane. Protein was transferred to PVDF membrane with a Trans-Blot Turbo Transfer System (Bio-Rad). Membranes were then blocked with 5% milk and 1% BSA in a buffer consisting of 150mM NaCl; 20mM Tris-HCl, pH 7.5; and 0.1% Tween for 1h. The blots were probed with antibodies against HA-tag (ab9110 (Abcam), 1:10 000 dilution) Citrate Synthase (#14309S (Cell Signaling Technology, Danvers, MA, USA), 1:1000 dilution), cleaved caspase-3 (#9661 (Cell Signaling Technology), 1:500 dilution) or total OXPHOS rodent Ab cocktail (#ab110413-MS604 (Abcam), 1:250 dilution) and incubated overnight at 4°C. Horseradish peroxidase (HRP)-conjugated goat anti-rabbit (#7074 (Cell Signaling Technology), 1:10 000 dilution), or goat anti-mouse (#1706516 (Bio-Rad), 1:2000 dilution) was used to detect the primary antibodies. Clarity Western ECL Substrate was used for visualization of proteins with a ChemiDoc XRS+ System (Bio-Rad). The signal intensity of

each protein band was measured with Image Lab software (version 5.2.1; Bio-Rad) and normalized to that of the total protein bands in the lane.

MTT assay

The viability of transduced INS1 β-cells was investigated with the Cell Proliferation Kit I (Roche, Basel, Switzerland), a MTT reagent kit, according to the manufacturer's instructions.

Caspase activity assay

Caspase-3 and -7 activity in transduced INS1 β-cells was measured with the Caspase-Glo® 3/7 assay (Promega, Madison, WI, USA) according to the manufacturer's instructions.

EdU incorporation assay

Proliferation was quantified using Click-iT™ Plus EdU Cell Proliferation Kit for Imaging (ThermoFisher Scientific). Cells were plated and transduced in 8-well chamber slides coated with poly-D-lysine. On the day of analysis, cells were exposed to 10µM of 5-ethynyl-2′-deoxyuridine (EdU) for 4h at 37°C and then fixed with 4% paraformaldehyde in PBS. EdU labelling was done according to the manufacturer's instructions. Images, 12 per well, were acquired in random locations of the wells with a confocal microscope and quantification was done manually with the Cell Counter Plugin in ImageJ to manually tag and count stained cells.

Transcription factor binding motif analysis

Analysis of transcription factor binding motif enrichment within promoter regions (-450bp - +50bp relative to the transcription start site) was performed by using Pscan (12) and the JASPAR database.

Transcriptomic profiling in Pax5-overexpressing INS1 β-cells

Total RNA quality was assessed by Agilent Technologies 2200 Tapestation and concentrations were measured by NanoDrop ND-1000 Spectrophotometer. 100ng of total RNA from Pax5-overexpressing (n=8) and control (GFP-overexpressing, n=8) INS1 β-cells was used in downstream analyses. GeneChip® WT Plus Reagent Kit (ThermoFisher Scientific) was used to generate amplified sense strand cDNA targets, fragmentation and labelling. 2.3µg of cDNA target was hybridized to Clariom™ S Rat Arrays for 16h at 45°C under rotation in Affymetrix Gene Chip Hybridization Oven 645 (ThermoFisher Scientific). Washing and staining was carried out on Affymetrix GeneChip® Fluidics Station 450 (ThermoFisher Scientific), according to the manufacturer's protocol. The fluorescent intensities were determined with Affymetrix GeneChip Scanner 3000 7G (ThermoFisher Scientific). Processing of raw data was performed in Transcriptome Analysis Console (TAC, v4.0) using quantile normalization and gene-level SST-RMA summarization. Sample group comparisons were performed using eBayes ANOVA tests, and p-values were corrected for multiple testing using the Benjamini-Hochberg method. Data were further processed to remove probes that corresponded to multiple genes and genes with outdated names/IDs. The R package biomaRt (Rnor_6.0 - Ensembl v. 104) was used to retrieve updated Ensembl gene names/IDs from the provided gene names,

NCBI Reference Sequence IDs, Entrez IDs or Ensembl transcript IDs. After this, probes that corresponded to more than 1 or no genes were discarded. Genes that corresponded to multiple probes, and therefore measured more than once, were represented by the probe with the highest absolute fold-change value between the sample groups.

Co-expression analysis

Co-expression networks among the DEGs were constructed using the R WGCNA package (v. 1.70-3) (13). The analysis was performed using the blockwiseModules function with parameters: power = 10, networkType = "signed", minModuleSize = 20, mergeCutHeight = 0.25, minKMEtoStay = 0, pamRespectsDendro = F. Default settings were selected for the rest of the parameters. First, a similarity matrix was constructed by calculating the correlation coefficient of each gene using Pearson's correlation. Here, a variance stabilizing transformation was applied to the gene expression values using Deseq2 and used as input. Considering only positively correlated genes as connected (signed network type and default by WGCNA), a power function was used to transform the similarity network into an adjacency matrix. After testing a range of power values and evaluating the corresponding scale independence and mean connectivity plots, a soft-thresholding power of 10 was selected to achieve a scale-free network with degree of independence above 0.80. Next, a topological overlap matrix was obtained from the adjacency matrix and hierarchical clustering using a dynamic tree cut algorithm was applied to identify co-expression gene clusters (modules).

Supplementary Tables

Table S1. Characteristics for donors of pancreatic islets included in the LUDC pancreatic islet cohort.

Variable	ND (n= 264)	T2D (n= 45)	P-value
Sex (M/F)	164/100	29/16	
Age (years)	58.7 ± 0.7 (19-86)	62.5 ± 1.4 (41-81)	0.033
BMI (kg/m²)	26.5 ± 0.3 (17.6-42.6)	28.4 ± 0.8 (20.1-50.9)	0.0064
HbaA1c (mmol/mol)	38.8 ± 0.3 (22.0-70.0)	51.4 ± 1.6 (38.0-86.0)	<0.0001
Stimulatory index	7.6 ± 0.4 (0.7-37.3)	5.1 ± 0.7 (0.7-21.2)	0.016

Data presented as mean ± SEM (min-max)

Data analysed by two-tailed t-test

ND: non-diabetic, T2D: type 2 diabetes

Stimulatory index is a measure of glucose-stimulated insulin secretion from the human islets cultured in vitro

Supplementary Figures

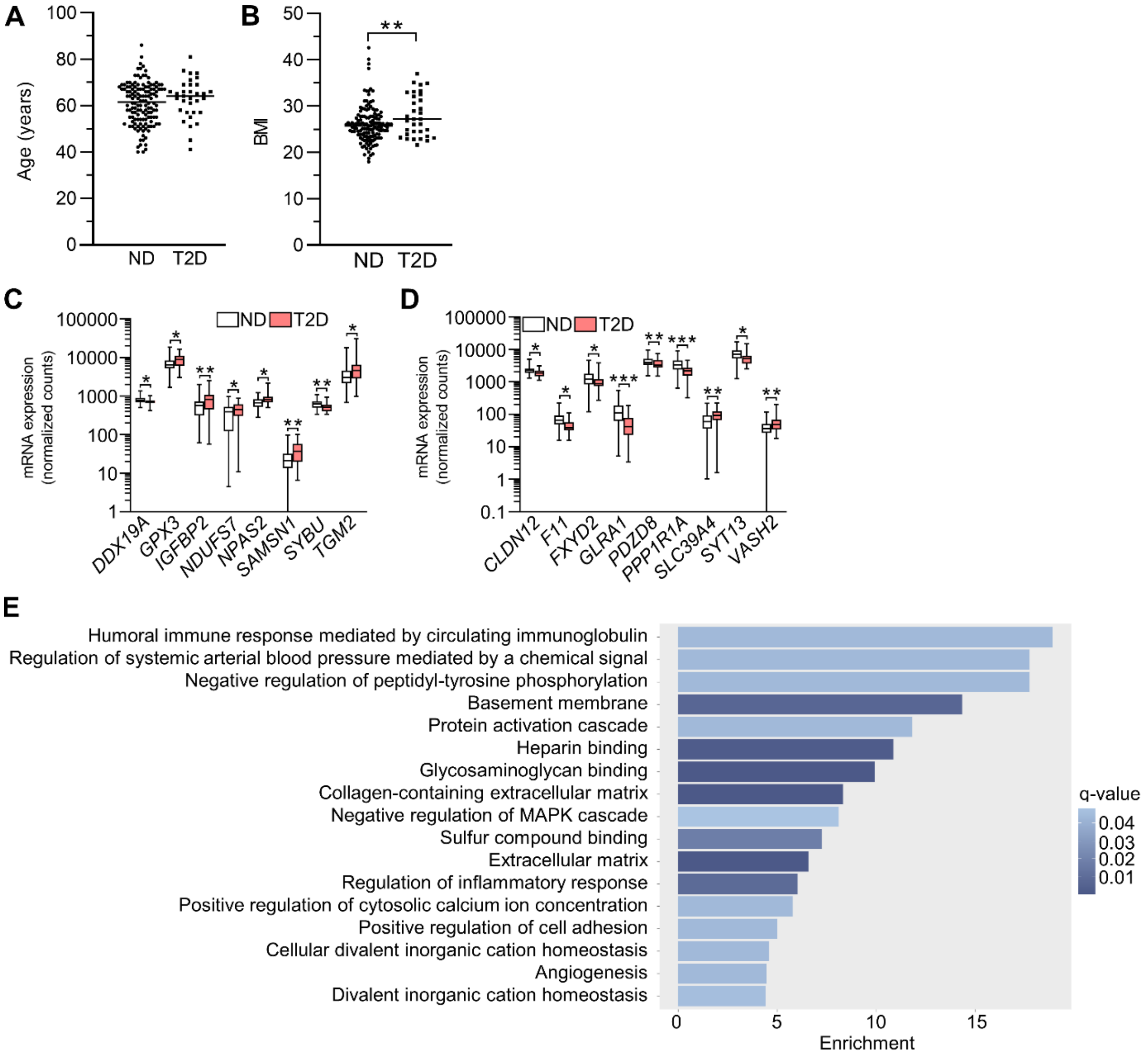

Figure S1. Donor characteristics and expression in human islets. (**A-B**) Graphs showing the age (**A**) and BMI (**B**) distribution in the *LUDC islet case-control cohort*. **p<0.01 based on a two-tailed t-test. The line shows the median. (**C-D**) Expression in the *LUDC islet case-control cohort* of the eight and nine genes that have previously been shown to be differentially expressed in α- (**C**) and β-cells (**D**), respectively, based on single cell RNA-sequencing (14-16). *q<0.05, **q<0.01, ***q<0.001, based on a generalized linear model as implemented in DESeq2 (4), with correction for age, sex, purity, and days in culture. (**E**) Gene ontology enrichment among the 90 islet DEGs that exhibit higher expression in α-cells compared to β-cells in the *LUDC sorted α/β-cell cohort*. Box-and-whisker plots show the median, 25th and 75th percentile, and minimum and maximum values.

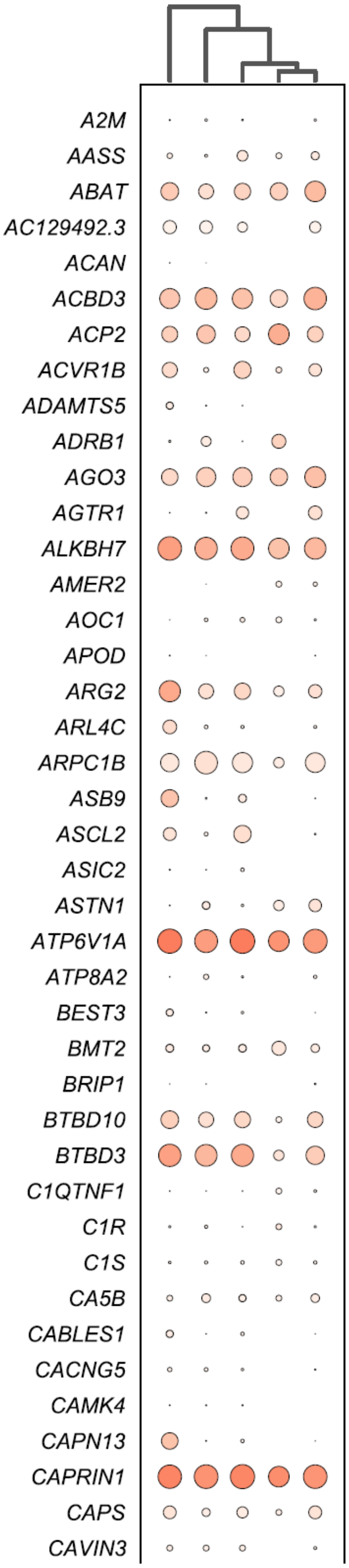
A2M
AASS
ABAT
AC129492.3
ACAN
ACBD3
ACP2
ACVR1B
ADAMTS5
ADRB1
AGO3
AGTR1
ALKBH7
AMER2
AOC1
APOD
ARG2
ARL4C
ARPC1B
ASB9
ASCL2
ASIC2
ASTN1
ATP6V1A
ATP8A2
BEST3
BMT2
BRIP1
BTBD10
BTBD3
C1QTNF1
C1R
C1S
CA5B
CABLES1
CACNG5
CAMK4
CAPN13
CAPRIN1
CAPS
CAVIN3

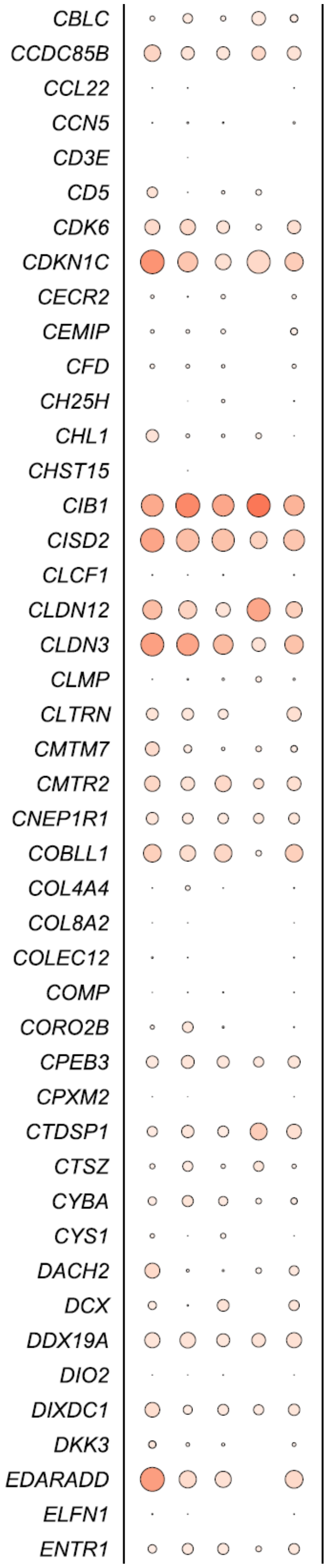
CBLC
CCDC85B
CCL22
CCN5
CD3E
CD5
CDK6
CDKN1C
CECR2
CEMIP
CFD
CH25H
CHL1
CHST15
CIB1
CISD2
CLCF1
CLDN12
CLDN3
CLMP
CLTRN
CMTM7
CMTR2
CNEP1R1
COBLL1
COL4A4
COL8A2
COLEC12
COMP
CORO2B
CPEB3
CPXM2
CTDSP1
CTSZ
CYBA
CYS1
DACH2
DCX
DDX19A
DIO2
DIXDC1
DKK3
EDARADD
ELFN1
ENTR1

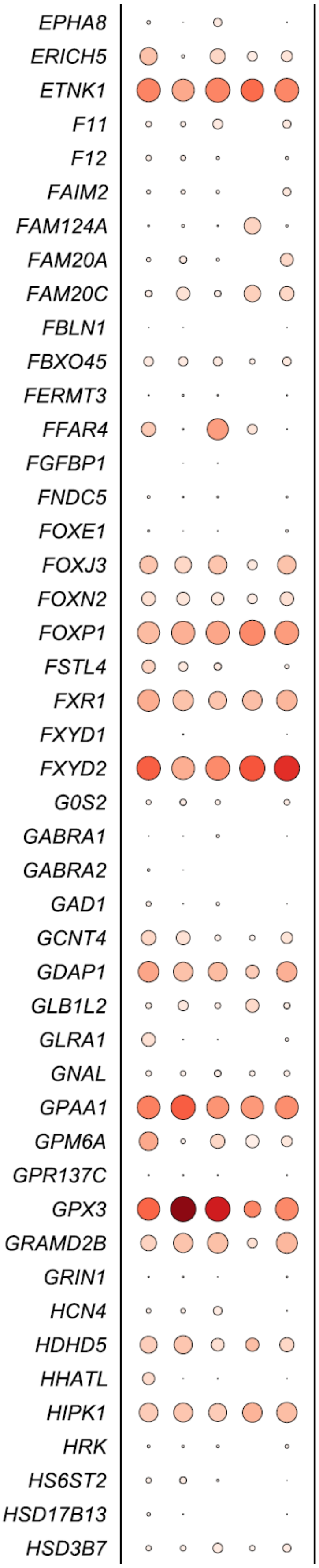
EPHA8
ERICH5
ETNK1
F11
F12
FAIM2
FAM124A
FAM20A
FAM20C
FBLN1
FBXO45
FERMT3
FFAR4
FGFBP1
FNDC5
FOXE1
FOXJ3
FOXN2
FOXP1
FSTL4
FXR1
FXYD1
FXYD2
G0S2
GABRA1
GABRA2
GAD1
GCNT4
GDAP1
GLB1L2
GLRA1
GNAL
GPAA1
GPM6A
GPR137C
GPX3
GRAMD2B
GRIN1
HCN4
HDHD5
HHATL
HIPK1
HRK
HS6ST2
HSD17B13
HSD3B7

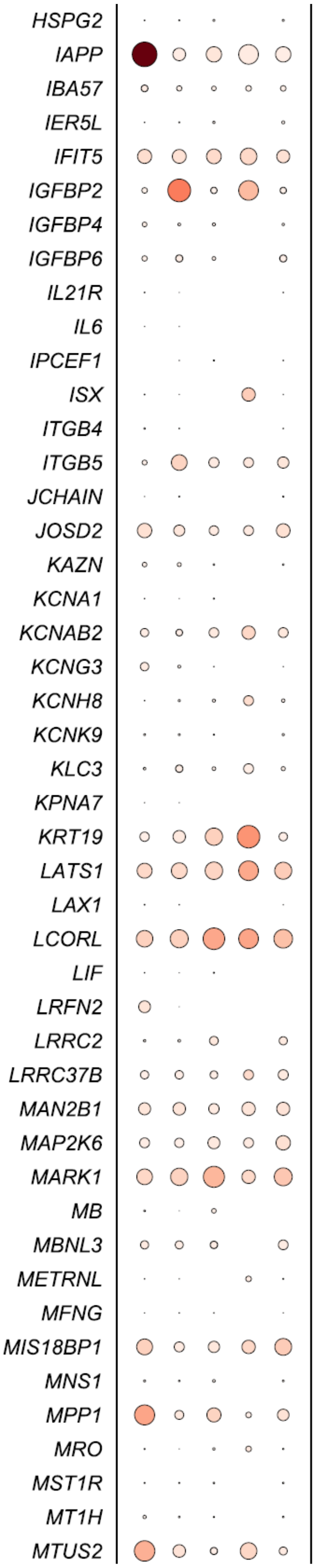
HSPG2
IAPP
IBA57
IER5L
IFIT5
IGFBP2
IGFBP4
IGFBP6
IL21R
IL6
IPCEF1
ISX
ITGB4
ITGB5
JCHAIN
JOSD2
KAZN
KCNA1
KCNAB2
KCNG3
KCNH8
KCNK9
KLC3
KPNA7
KRT19
LATS1
LAX1
LCORL
LIF
LRFN2
LRRC2
LRRC37B
MAN2B1
MAP2K6
MARK1
MB
MBNL3
METRNL
MFNG
MIS18BP1
MNS1
MPP1
MRO
MST1R
MT1H
MTUS2

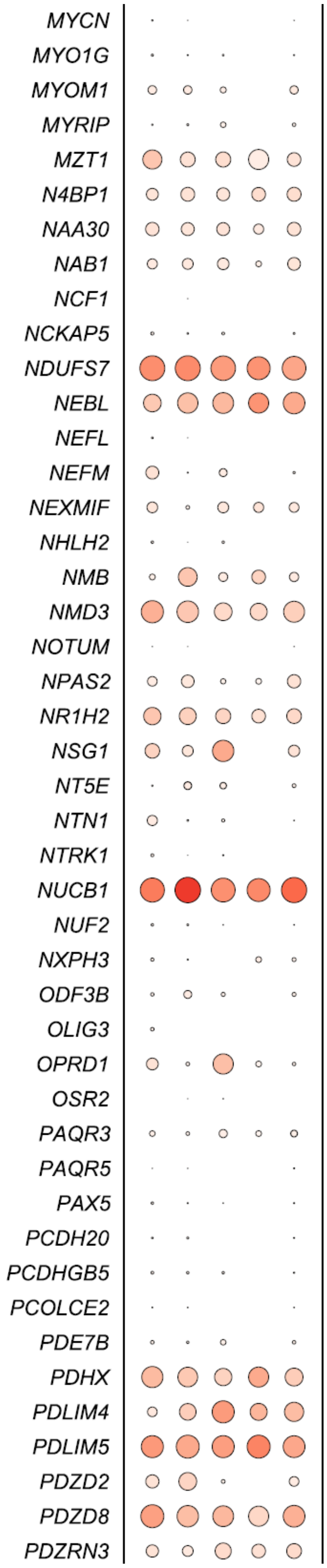
MYCN
MYO1G
MYOM1
MYRIP
MZT1
N4BP1
NAA30
NAB1
NCF1
NCKAP5
NDUFS7
NEBL
NEFL
NEFM
NEXMIF
NHLH2
NMB
NMD3
NOTUM
NPAS2
NR1H2
NSG1
NT5E
NTN1
NTRK1
NUCB1
NUF2
NXPH3
ODF3B
OLIG3
OPRD1
OSR2
PAQR3
PAQR5
PAX5
PCDH20
PCDHGB5
PCOLCE2
PDE7B
PDHX
PDLIM4
PDLIM5
PDZD2
PDZD8
PDZRN3

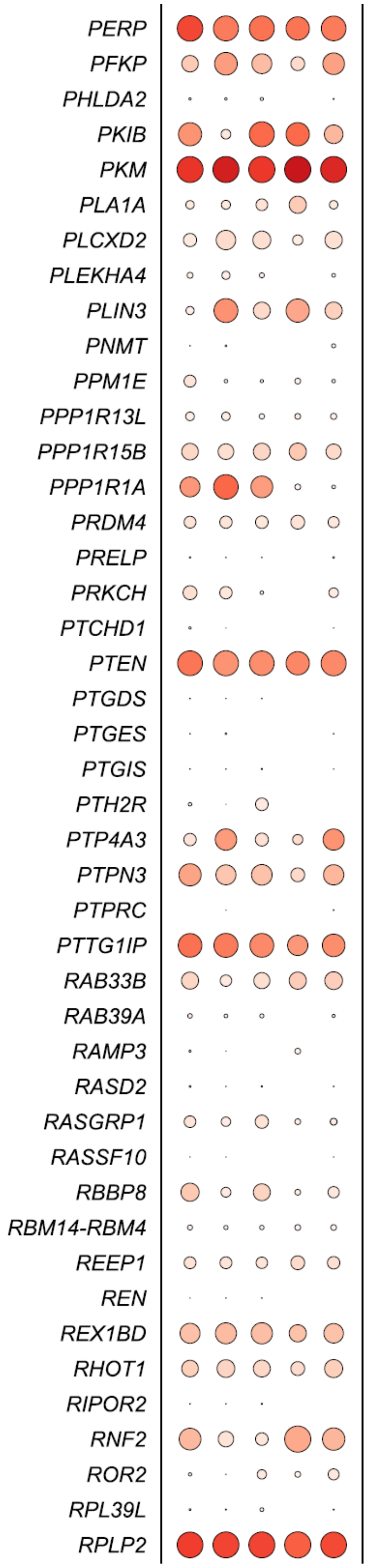
PERP
PFKP
PHLDA2
PKIB
PKM
PLA1A
PLCXD2
PLEKHA4
PLIN3
PNMT
PPM1E
PPP1R13L
PPP1R15B
PPP1R1A
PRDM4
PRELP
PRKCH
PTCHD1
PTEN
PTGDS
PTGES
PTGIS
PTH2R
PTP4A3
PTPN3
PTPRC
PTTG1IP
RAB33B
RAB39A
RAMP3
RASD2
RASGRP1
RASSF10
RBBP8
RBM14-RBM4
REEP1
REN
REX1BD
RHOT1
RIPOR2
RNF2
ROR2
RPL39L
RPLP2

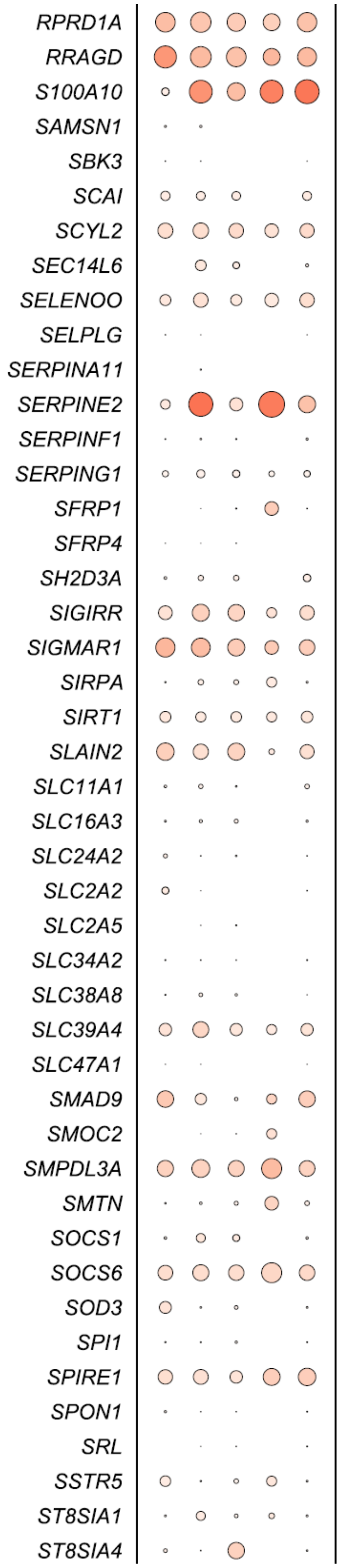
RPRD1A
RRAGD
S100A10
SAMSN1
SBK3
SCAI
SCYL2
SEC14L6
SELENOO
SELPLG
SERPINA11
SERPINE2
SERPINF1
SERPING1
SFRP1
SFRP4
SH2D3A
SIGIRR
SIGMAR1
SIRPA
SIRT1
SLAIN2
SLC11A1
SLC16A3
SLC24A2
SLC2A2
SLC2A5
SLC34A2
SLC38A8
SLC39A4
SLC47A1
SMAD9
SMOC2
SMPDL3A
SMTN
SOCS1
SOCS6
SOD3
SPI1
SPIRE1
SPON1
SRL
SSTR5
ST8SIA1
ST8SIA4

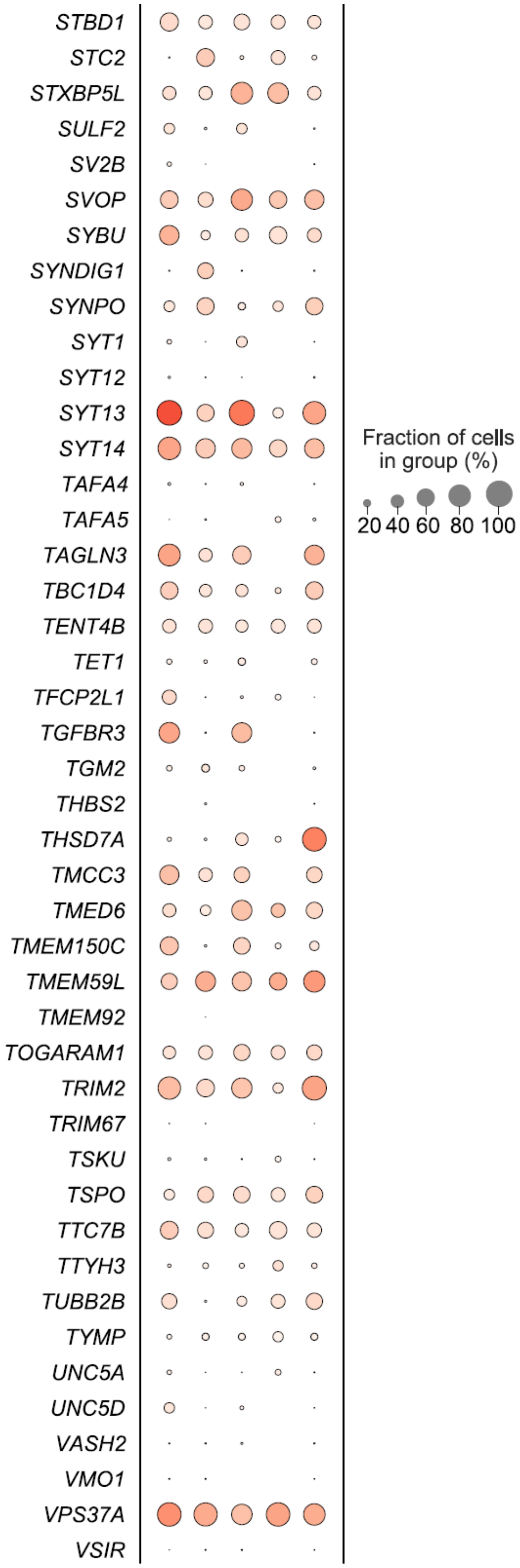
STBD1
STC2
STXBP5L
SULF2
SV2B
SVOP
SYBU
SYNDIG1
SYNPO
SYT1
SYT12
SYT13
SYT14
TAFA4
TAFA5
TAGLN3
TBC1D4
TENT4B
TET1
TFCP2L1
TGFBR3
TGM2
THBS2
THSD7A
TMCC3
TMED6
TMEM150C
TMEM59L
TMEM92
TOGARAM1
TRIM2
TRIM67
TSKU
TSPO
TTC7B
TTYH3
TUBB2B
TYMP
UNC5A
UNC5D
VASH2
VMO1
VPS37A
VSIR
Fraction of cells in group (%)
20 40 60 80 100

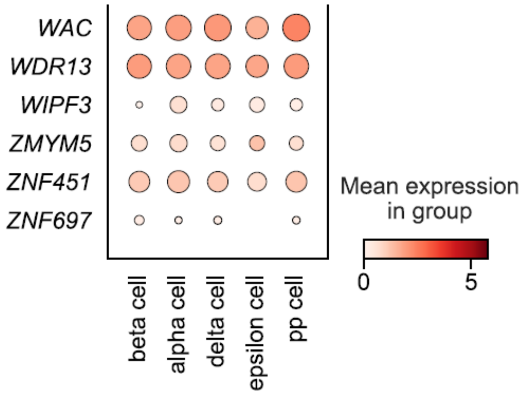

Figure S2. Most T2D DEGs are expressed in β-cells and the other endocrine islet cell types. Dot blow showing the expression of the 395 DEGs identified in the LUDC islet case-control cohort in the different endocrine islet cell types. The figure is based on sc-RNA sequencing data on human pancreatic islets from Segerstolpe et al (15) and only genes with a normalized expression above 0.0 in at least three single cells are included.

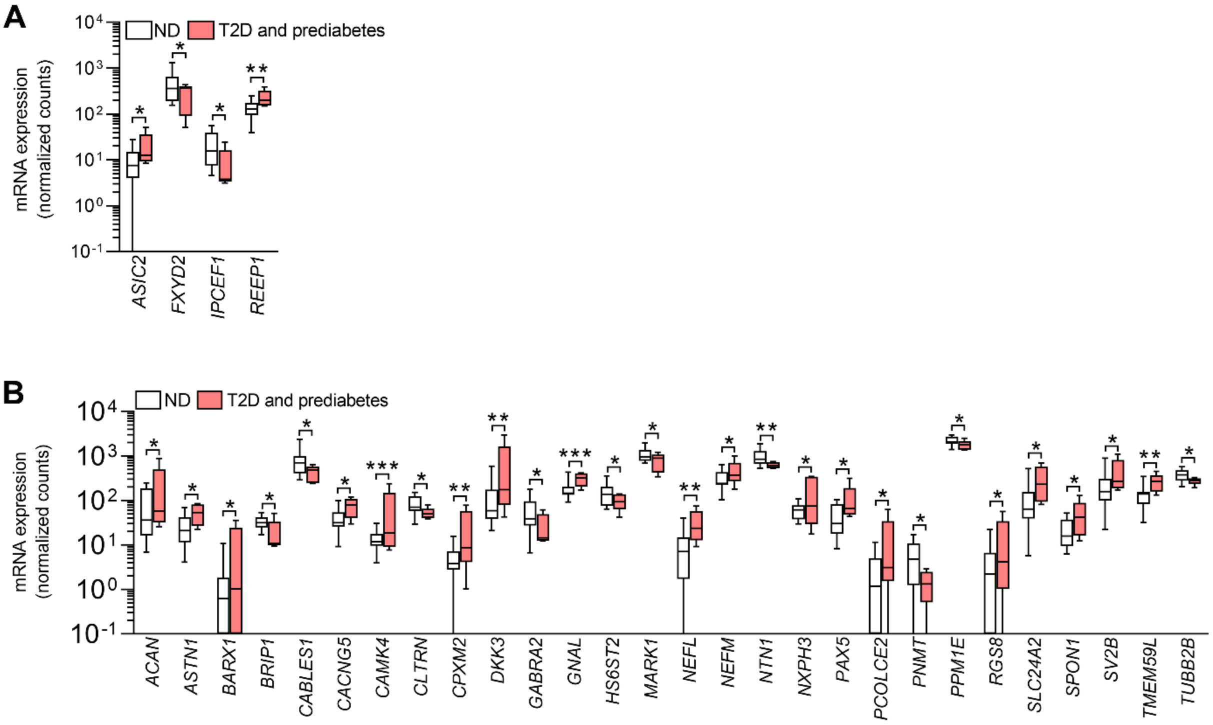

Figure S3. Gene expression in sorted α- and β-cells. (**A-B**) Expression in sorted α- (**A**) and β-cells (**B**) of genes that are differentially expressed in the same direction in T2D in islets of the *LUDC islet case-control cohort* as well as in prediabetes/T2D in α- or β-cells of the *LUDC sorted α/β-cell cohort*. *p<0.05, **p<0.01, ***p<0.001, based on a generalized linear model as implemented in DESeq2 (4). Box-and-whisker plots show the median, 25th and 75th percentile, and minimum and maximum values.

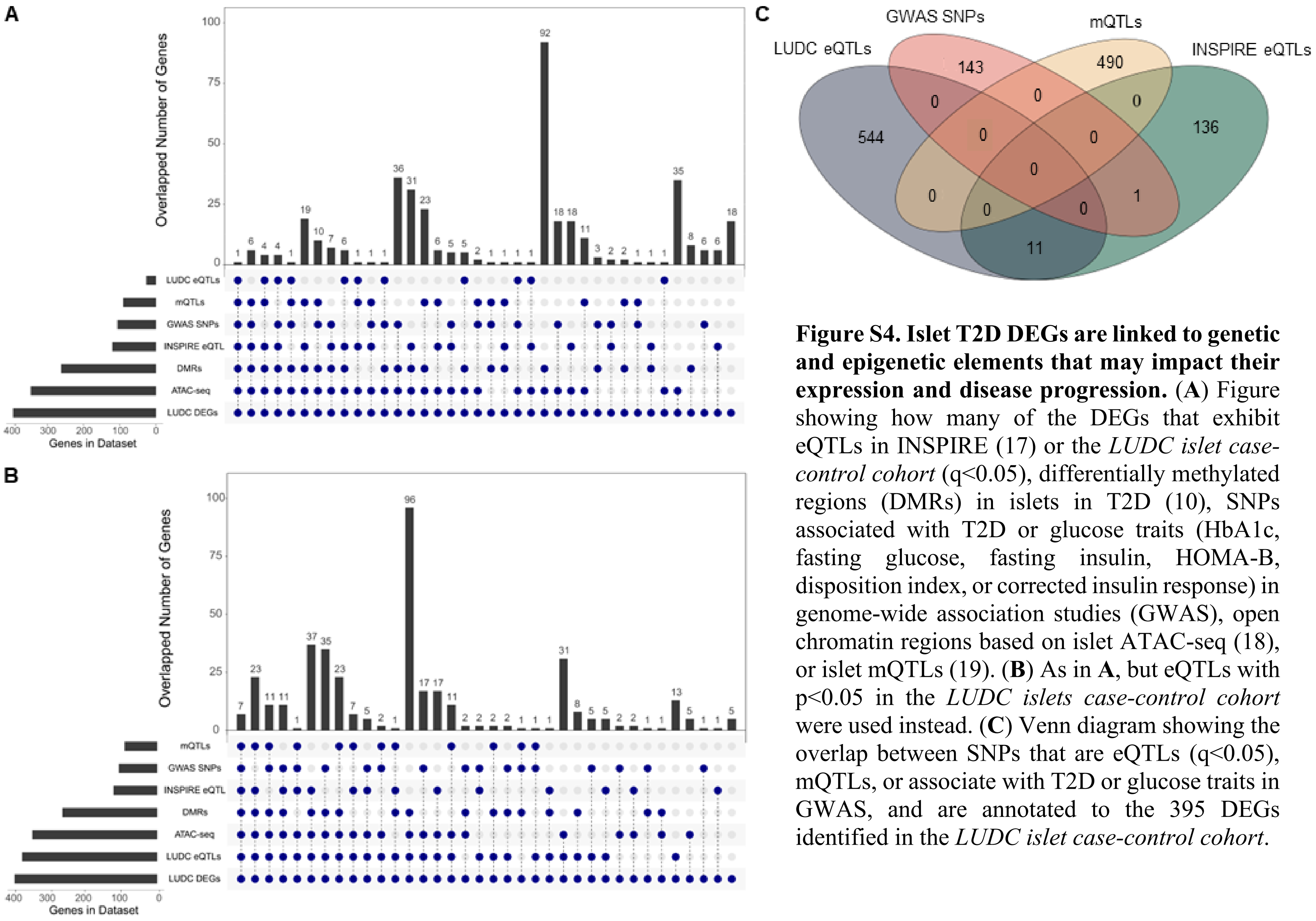

Figure S4. Islet T2D DEGs are linked to genetic and epigenetic elements that may impact their expression and disease progression. (**A**) Figure showing how many of the DEGs that exhibit eQTLs in INSPIRE (17) or the *LUDC islet case-control cohort* (q<0.05), differentially methylated regions (DMRs) in islets in T2D (10), SNPs associated with T2D or glucose traits (HbA1c, fasting glucose, fasting insulin, HOMA-B, disposition index, or corrected insulin response) in genome-wide association studies (GWAS), open chromatin regions based on islet ATAC-seq (18), or islet mQTLs (19). (**B**) As in **A**, but eQTLs with p<0.05 in the *LUDC islets case-control cohort* were used instead. (**C**) Venn diagram showing the overlap between SNPs that are eQTLs (q<0.05), mQTLs, or associate with T2D or glucose traits in GWAS, and are annotated to the 395 DEGs identified in the *LUDC islet case-control cohort*.

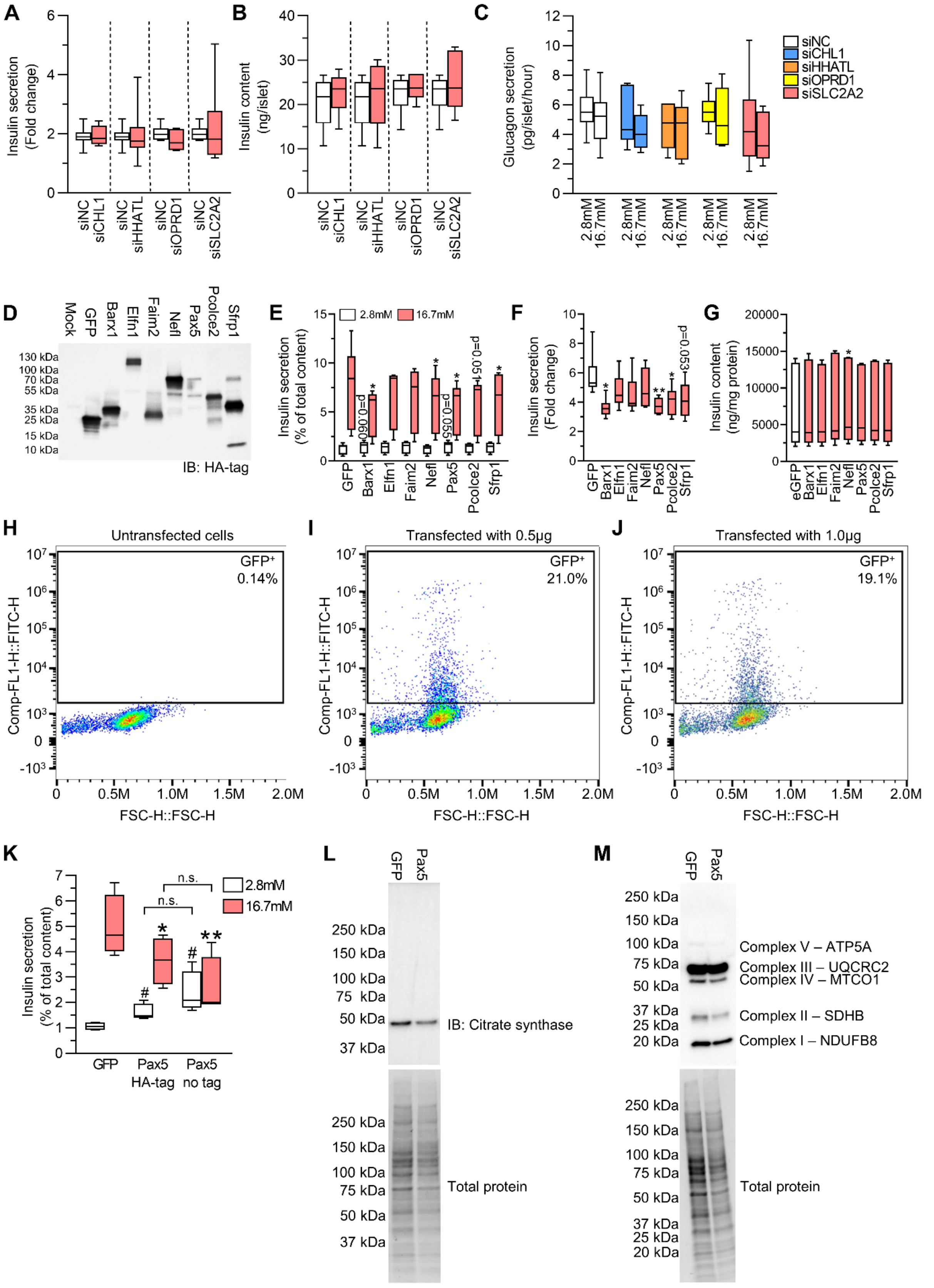

Figure S5. Genes that exhibit differential gene expresison in islets from individuals with T2D affect β-cell metabolism and insulin secretion. (**A-C**) Insulin secretion expressed as fold change (**A**), insulin content (**B**), and glucagon secrtion (**C**) in human islets transfected with the indicated siRNA. (**D**) Western blot showing the expression of GFP, Barx1, Elfn1, Faim2, Nefl,

Pax5, Pcolce2, and Sfrp1 in plasmid transfected 832/13 INS1 β-cells. (**E-G**) Effect of overexpression on insulin secretion expressed in absolute values (**E**) and fold change (**F**), and total insulin content (**G**) in transfected INS1 β-cells. E: *$p<0.05$ vs GFP at 16.7mM glucose, based on paired t-tests. F-G: *$p<0.05$, **$p<0.01$ vs GFP, based on paired t-tests. (**H-J**) Number of GFP^+ cells after plasmid transfection with 0 (**H**), 0.5 (**I**), or 1.0ug (**J**) plasmid/well. (**K**) Overexpression of both HA-tagged and untagged Pax5 impairs insulin secretion in INS1 β-cells. *$p<0.05$, **$p<0.01$ vs GFP at 16.7mM glucose, $^{\#}p<0.05$ vs GFP at 2.8mM glucose, based on paired t-tests. (**L-M**) Western blot showing levels of citrate synthase (**L**) and subunits of complex I-V of the electron transport chain (**M**) in Pax5-overexpressing INS1 β-cells. Box-and-whisker plots show the median, 25th and 75th percentile, and minimum and maximum values.

References

1. Patro R, Duggal G, Love MI, Irizarry RA, and Kingsford C. Salmon provides fast and bias-aware quantification of transcript expression. Nat Methods. 2017;14(4):417-9.

2. Ewels P, Magnusson M, Lundin S, and Kaller M. MultiQC: summarize analysis results for multiple tools and samples in a single report. Bioinformatics. 2016;32(19):3047-8.

3. Soneson C, Love MI, and Robinson MD. Differential analyses for RNA-seq: transcript-level estimates improve gene-level inferences. F1000Res. 2015;4:1521.

4. Love MI, Huber W, and Anders S. Moderated estimation of fold change and dispersion for RNA-seq data with DESeq2. Genome Biol. 2014;15(12):550.

5. Hrvatin S, Deng F, O'Donnell CW, Gifford DK, and Melton DA. MARIS: method for analyzing RNA following intracellular sorting. PLoS One. 2014;9(3):e89459.

6. Purcell S, Neale B, Todd-Brown K, Thomas L, Ferreira MA, Bender D, et al. PLINK: a tool set for whole-genome association and population-based linkage analyses. Am J Hum Genet. 2007;81(3):559-75.

7. Munoz-Fuentes V, Cacheiro P, Meehan TF, Aguilar-Pimentel JA, Brown SDM, Flenniken AM, et al. The International Mouse Phenotyping Consortium (IMPC): a functional catalogue of the mammalian genome that informs conservation. Conserv Genet. 2018;19(4):995-1005.

8. Rozman J, Rathkolb B, Oestereicher MA, Schutt C, Ravindranath AC, Leuchtenberger S, et al. Identification of genetic elements in metabolism by high-throughput mouse phenotyping. Nat Commun. 2018;9(1):288.

9. Hohmeier HE, Mulder H, Chen G, Henkel-Rieger R, Prentki M, and Newgard CB. Isolation of INS-1-derived cell lines with robust ATP-sensitive K+ channel-dependent and -independent glucose-stimulated insulin secretion. Diabetes. 2000;49(3):424-30.

10. Volkov P, Bacos K, Ofori JK, Esguerra JL, Eliasson L, Ronn T, et al. Whole-Genome Bisulfite Sequencing of Human Pancreatic Islets Reveals Novel Differentially Methylated Regions in Type 2 Diabetes Pathogenesis. Diabetes. 2017;66(4):1074-85.

11. Wierup N, Svensson H, Mulder H, and Sundler F. The ghrelin cell: a novel developmentally regulated islet cell in the human pancreas. Regul Pept. 2002;107(1-3):63-9.

12. Zambelli F, Pesole G, and Pavesi G. Pscan: finding over-represented transcription factor binding site motifs in sequences from co-regulated or co-expressed genes. Nucleic Acids Res. 2009;37(Web Server issue):W247-52.

13. Langfelder P, and Horvath S. WGCNA: an R package for weighted correlation network analysis. BMC Bioinformatics. 2008;9:559.

14. Lawlor N, George J, Bolisetty M, Kursawe R, Sun L, Sivakamasundari V, et al. Single-cell transcriptomes identify human islet cell signatures and reveal cell-type-specific expression changes in type 2 diabetes. Genome Res. 2017;27(2):208-22.

15. Segerstolpe A, Palasantza A, Eliasson P, Andersson EM, Andreasson AC, Sun X, et al. Single-Cell Transcriptome Profiling of Human Pancreatic Islets in Health and Type 2 Diabetes. Cell Metab. 2016;24(4):593-607.

16. Xin Y, Kim J, Okamoto H, Ni M, Wei Y, Adler C, et al. RNA Sequencing of Single Human Islet Cells Reveals Type 2 Diabetes Genes. Cell Metab. 2016;24(4):608-15.

17. Vinuela A, Varshney A, van de Bunt M, Prasad RB, Asplund O, Bennett A, et al. Genetic variant effects on gene expression in human pancreatic islets and their implications for T2D. Nat Commun. 2020;11(1):4912.

18. Bysani M, Agren R, Davegardh C, Volkov P, Ronn T, Unneberg P, et al. ATAC-seq reveals alterations in open chromatin in pancreatic islets from subjects with type 2 diabetes. Sci Rep. 2019;9(1):7785.

19. Olsson AH, Volkov P, Bacos K, Dayeh T, Hall E, Nilsson EA, et al. Genome-wide associations between genetic and epigenetic variation influence mRNA expression and insulin secretion in human pancreatic islets. PLoS Genet. 2014;10(11):e1004735.
